# Nanofiber-mediated sequential photothermal antibacteria and macrophage polarization for healing MRSA-infected diabetic wounds

**DOI:** 10.1186/s12951-021-01152-4

**Published:** 2021-12-05

**Authors:** Zhou Xu, Bin Deng, Xuewen Wang, Jie Yu, Zhuobin Xu, Penggang Liu, Caihong Liu, Yuan Cai, Fei Wang, Rongling Zong, Zhiling Chen, Hua Xing, Gang Chen

**Affiliations:** 1 Institute of Comparative Medicine, College of Veterinary Medicine, Jiangsu Co-Innovation Center for Prevention and Control of Important Animal Infectious Diseases and Zoonoses, Joint International Research Laboratory of Agriculture and Agri-Product Safety, The Ministry of Education of China, Yangzhou University, Yangzhou, 225009 China; 2 Department of Gastroenterology, Affiliated Hospital, Yangzhou University, Yangzhou, 225009 China; 3 Department of Traditional Chinese Medicine, Affiliated Hospital, Yangzhou University, Yangzhou, 225009 China; 4 Institute of Translational Medicine, Medical College, Yangzhou University, Yangzhou, 225001 China; 5 School of Basic Medical Sciences, Xuzhou Medical University, Xuzhou, 221004 China

**Keywords:** Nanofibers, Polydopamine, Antibacteria, Macrophage polarization, Infected diabetic wounds

## Abstract

**Background:**

Diabetic wound healing remains a challenge because of its susceptibility to drug-resistant bacterial infection and its persistent proinflammatory state. Switching from proinflammatory M1 macrophages (Mφs) to proregenerative M2 dominant Mφs in a timely manner accelerates wound healing by coordinating inflammatory, proliferative, and angiogenic processes.

**Methods:**

We propose a sequential photothermal antibacterial and subsequent M2 Mφ polarization strategy based on nanofibers (NFs) consisting of polydopamine (PDA) coating on curcumin (Cur) nanocrystals to treat Methicillin-resistant *Staphylococcus aureus* (MRSA)-infected diabetic wounds.

**Results:**

The PDA/Cur NFs showed excellent photothermal conversion and antibacterial effects due to the PDA shell under laser irradiation, consequently resulting in the release of the inner Cur with the ability to promote cell proliferation and reinforce the M2 Mφ phenotype in vitro. In vivo studies on MRSA-infected diabetic wounds showed that PDA/Cur NFs not only inhibited MRSA infection but also accelerated the wound regeneration process. Furthermore, the NFs displayed the ability to promote the M2 Mφ phenotype with enhanced collagen deposition, angiogenesis, and cell proliferation.

**Conclusion:**

Overall, the NFs displayed great potential as promising therapeutics for healing infected diabetic wounds through a sequential photothermal antibacterial and M2 Mφ polarization strategy.

**Graphical abstract:**

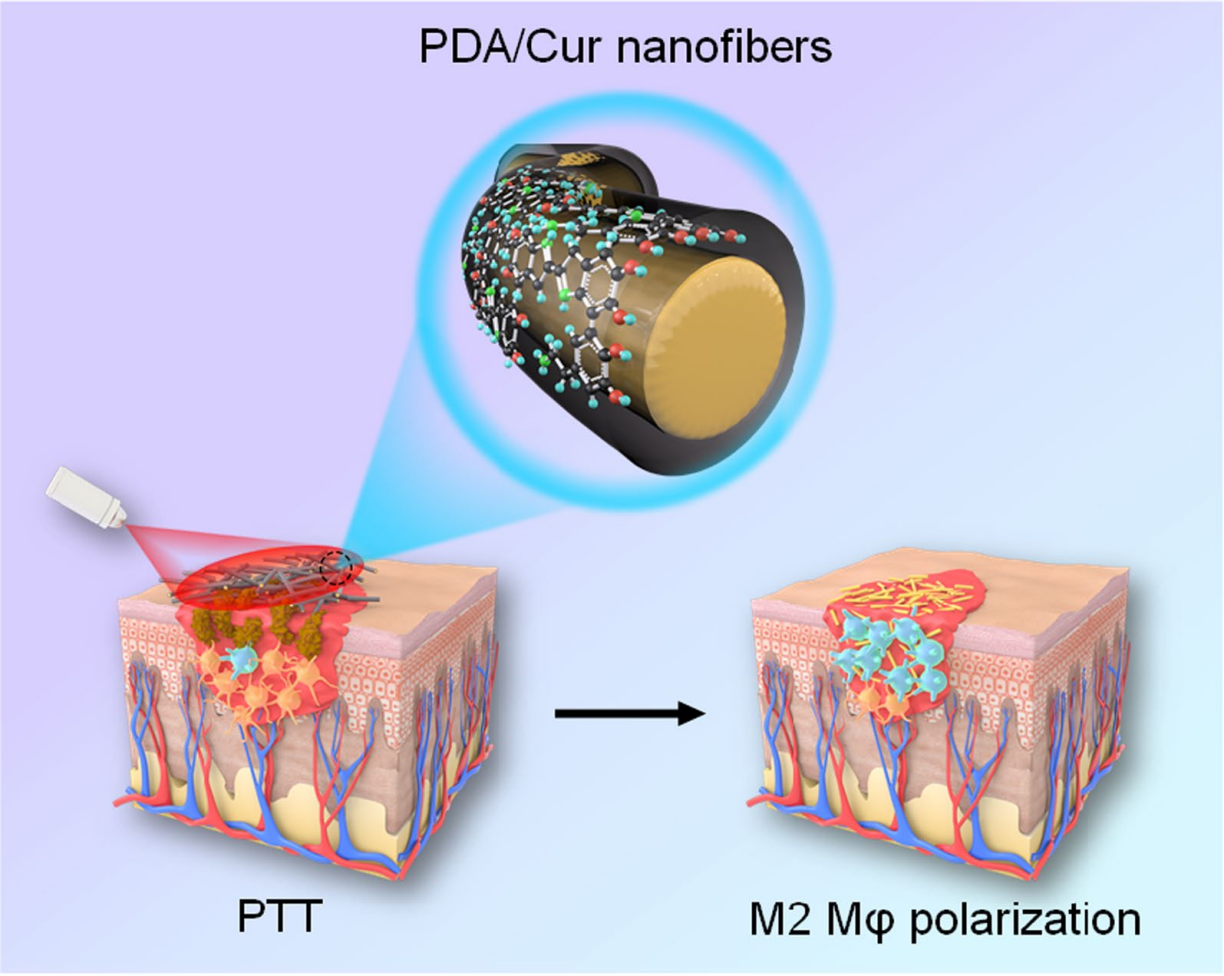

**Supplementary Information:**

The online version contains supplementary material available at 10.1186/s12951-021-01152-4.

## Introduction

Impaired wounds in diabetic patients are difficult to heal which seriously threatens public health [1–4]. Diabetic wounds are clinically chronic complications, and the vulnerability to bacterial infection, especially drug-resistant bacteria, will hinder wound healing in local microenvironments [5–12]. Methicillin-resistant *Staphylococcus aureus* (MRSA), a representative drug-resistant strain with high morbidity and mortality, has spread globally and is often found in the wounds of patients with diabetes [13, 14]. To combat drug-resistant bacteria, increasing antibiotic dosage is the most common approach in clinical practice, which results in severe side effects and contributes to the evolution of drug-resistant microbes in feedback. Eradicating bacteria should be the first step in the process of healing infected wounds; therefore, the application of efficient strategies to treat drug-resistant infections is urgently necessary.

Photothermal therapy (PTT) has attracted significant attention in antibacterial and antitumor applications because of its high selectivity, low invasiveness, and minor side effects [15–21]. Upon exposure to laser irradiation, photothermal agents can generate local hyperthermia to kill pathogens with high efficiency and, unlike antibiotic therapy, the development of drug resistance can be avoided in PTT [22]. So far, a variety of photothermal antibacterial agents have been developed, such as carbon-based nanoparticles (NPs) [23], Au-based NPs [24], Ag-based NPs [25], Cu-based NPs [26], graphene-based NPs [27], and organic dyes [28], etc. However, their inherent biosafety and long-term toxicity, low photothermal conversion efficiency, and high cost limit their further application in vivo and in clinical practice. Compared with the above NPs, polydopamine (PDA), a mussel-inspired material, possesses the advantages of easy fabrication, good biocompatibility, and excellent photothermal performance, which render them great candidates for antibacterial therapy [29–31].

There has been increasing evidence that delayed transition from the inflammatory phase to proliferation limits the recovery of diabetic wounds. In addition, bacterial infection in diabetic wounds aggravates the inflammatory state and impedes wound closure. Macrophages (Mφs) play critical roles in modulating the inflammatory-proliferation phase transition, which exert dichotomous functions in the wound environment. During the inflammation phase, the M1 phenotype at the wound site can remove damaged tissues. Under healthy conditions, Mφs then differentiate into anti-inflammatory and proregenerative M2 phenotypes, which enter the proliferative phase by preventing excess inflammation, promoting stromal cell proliferation, and angiogenesis. Unfortunately, under pathological conditions, such as diabetes or bacterial infection, the transition of Mφs from M1 to M2 phenotype fails to occur, and the function of M1 Mφs is prolonged and persists [32, 33]. Therefore, it is necessary to reprogram Mφs toward the M2 state at appropriate time points in diabetic wound therapy [34]. The most common strategy based on Mφ modulation in wound healing is the supplementation of exogenous M2 Mφs at the wound site [35]. However, the remaining proinflammatory M1 Mφs are believed to destroy skin integrity and function [36–38]. Curcumin (Cur), a natural polyphenol extracted from turmeric, is a well-known wound-healing agent with proven anti-inflammatory effects [39–43]. Cur has also been reported to have the ability to polarize Mφ from the M1 to M2 phenotype, making it a potential therapeutic agent for healing diabetic wounds [44–46].

**Scheme 1:**
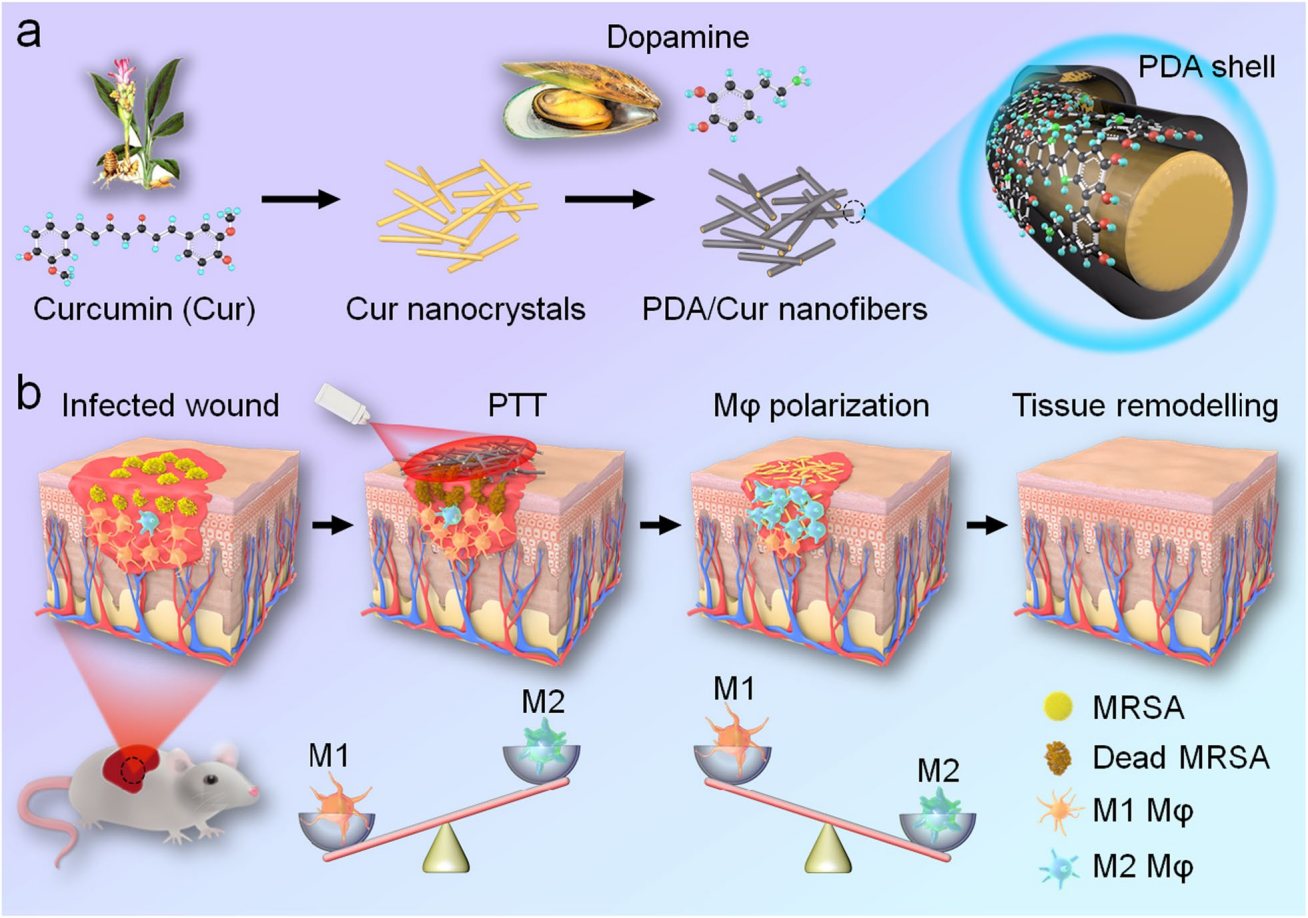
Fabrication and mechanism of action of PDA/Cur NFs. **a** Preparation of PDA/Cur NFs. **b** Schematic illustration for the mechanism that photothermal antibacteria and M2 Mφ polarization for healing MRSA-infected diabetic wounds

To simultaneously deal with bacterial infection and disordered Mφ differentiation throughout the wound infection process, we orchestrated a sequential therapy strategy depending on the initial killing bacteria and subsequent M2 Mφ polarization. We first constructed nanofibers (NFs) consisting of Cur nanocrystals coated with degradable PDA nanotubes (NTs) (Scheme 1). The outer PDA aims to show photothermal conversion and antibacterial effects upon laser irradiation, thereby promoting the release of the inner Cur to modulate the Mφ phenotype programmatically. The morphology, physicochemical properties, photothermal effects, and antibacterial activities of the NFs were assessed. In addition, cytotoxicity, cell migration, and Mφ polarization were evaluated. Finally, an MRSA-infected diabetic wound model was established and the sequential therapeutic effects of NFs were investigated in vivo.

## Materials and methods

### Materials

Dopamine (DA) hydrochloride (98%) and tris hydrochloride (Tris-HCl) were purchased from Aladdin Industrial Inc. (Shanghai, China). Cur, lipopolysaccharide (LPS), 3-[4,5-diamethylthiazol-2-yl]2,5-diphenyltetrazoliumbromide (MTT), and streptozotocin (STZ) were purchased from Sigma-Aldrich Industrial Inc. (St. Louis, Missouri, USA). Dulbecco’s Modified Eagle’s Medium (DMEM) and fetal bovine serum (FBS) were obtained from Gibco BRL Life Technologies Inc. (Carlsbad, CA, USA). Penicillin, streptomycin, crystal violet, and 4, 6-diamidino-2-phenylindole (DAPI) staining solution were got from Solarbio Industrial Inc. (Shanghai, China). PE-conjugated CD86 monoclonal antibody, PE-conjugated CD80 monoclonal antibody, PE-conjugated CD206 monoclonal antibody, and APC-conjugated CD11b monoclonal antibody were purchased from Invitrogen (Carlsbad, USA). Propidium iodide (PI) were obtained from Beyotime Biotechnology (Shanghai, China). SYTO™ 9 green fluorescent nucleic acid stain was ordered from Thermo Fisher Scientific (Waltham, Massachusetts, USA). Murine macrophage cell line RAW264.7 and murine fibroblast cell line L929 were got from Cell Bank of the Chinese Academic of Sciences (Shanghai, China). Drug-resistant *S. aureus* ATCC 43300 (MRSA) was from Dr. Xu (School of Medicine, Yangzhou University).

### Synthesis and characterization of PDA/Cur NFs

In brief, 50 mg of Cur was dissolved in 50 mL ethanol, and then 200 mL of water was added. Because Cur is insoluble in water, the solvent exchange induces the separation of the curcumin nanocrystals, collected by centrifugation at 12,000 rpm for 10 min, and the product was lyophilized for 24 h to obtain Cur nanocrystal powder. Cur nanocrystals (10 mg) were dispersed in 50 mL of Tris-HCl (10 mmol, pH 8.5) and 50 mg of DA hydrochloride was added, and the solution was stirred for 12 h, and the PDA/Cur NFs were collected by centrifugation at 12,000 rpm for 10 min. The products were lyophilized to obtain the PDA/Cur NF powder. PDA NTs were prepared by dissolving Cur nanocrystals in ethanol [47].

Scanning electron microscopy (SEM) images were acquired using a scanning electron microscope S-4800 II (Hitachi, Japan). Transmission electron microscopy (TEM) images were obtained using TEM Tecnai 12 transmission electron microscope (Philips, Netherlands). The Raman shift was measured using a DXR^TM^3xi Raman imaging microscope (Thermo Fisher, USA). Fourier-transform infrared (FTIR) spectrum was performed using a Fourier transform infrared spectrometer (Bruker, Germany), and FTIR spectra were recorded using KBr pellets at 298 K in the range of 4000–400 cm^−1^. Dried KBr powder (100 mg) was placed in an agate mortar, and PDA/Cur NFs (2 mg) was added and ground under infrared light. The ground sample was carefully placed in the tablet press. The handle was cranked to increase the pressure to 0.8 MPa and hold the pressure for 1 min. Then the air valve was opened and taken out the KBr sheet. Finally, the samples were determined by Fourier Transform infrared spectrometer. The FTIR spectrum of DA, Cur, PDA NTs was recorded with the same method. X-ray diffraction (XRD) was performed using an X-ray diffractometer D8 ADVANCE (Bruker, Germany). X-ray photoelectron spectroscopy (XPS) was performed using an ESCALAB by X-ray photoelectron spectrometer ESCALAB 250Xi (Thermo Scientific). Elemental analysis of the PDA/Cur NFs was performed using HTREM Tecnai G2 F30 S-TWIN (FEI, USA). The UV–Vis-NIR spectra were measured by a UV spectrometer Cary 5000 (Varian, USA). The Cur loading capacity of the PDA/Cur NFs was also measured using a UV spectrometer Cary 5000 (Varian, USA). PDA/Cur NFs (1 mg) was dissolved in 10 mL ethanol and ultrasonicated for 10 min, and the supernatant was then measured using a UV spectrometer at a wavelength of 425 nm. Cur release from NFs was detected by reversed-phase high-performance liquid chromatography. First, the PDA/Cur NF suspension (1 mg/mL, 1 mL) was transferred to dialysis bags (the interception molecular weight was 3000), which was then immersed in 10 mL of release medium (0.01 M PBS, pH 6.5 or pH 7.4) at 37 °C. Laser irradiation and 500 μL of medium were taken at predetermined time intervals and compensated with an equal volume of fresh medium. The released Cur content was measured using HPLC. HPLC details are shown as follows: The HPLC system was Waters E2695 (Waters, USA). A reversed-phase C18 column (4.6 × 150 mm, particle size 5 μm, Eclipse XDB, Agilent, Palo Alto, CA, USA) was used for HPLC separation. The mobile phase was programmed with A: B ratios of 50:50, where A denotes 0.05% acetic acid and B denotes acetonitrile for 3 min. Before use, the mobile phase was filtered through a 0.45 μm Millipore membrane filter and degassed by sonication 2510R-DTH (Bransonic, CT, USA). The flow rate used was 1 mL/min. The system was allowed to stabilize for 2 min between consecutive injections, and the column temperature was maintained at 25 °C. Detection was performed at a wavelength of 425 nm, and the sample injection volume was 10 μL. A calibration curve with individual standards and the sample was used to quantify the Cur concentrations.

### Photothermal effects PDA/Cur NFs

The photothermal performance of PDA/Cur NFs (200 μg/mL) and PDA NTs (180 μg/mL) exposed to a near-infrared (NIR) laser (808 nm, 0.8 W/cm^2^) for 300 s was recorded using a near-infrared imager at intervals of 60 s. Then, the photothermal conversion effects were determined using a temperature detector. Different concentrations of PDA/Cur NFs (50, 100, 150, 200 μg/mL) and PDA NTs (45, 90, 135, 180 μg/mL) were exposed to a NIR laser (808 nm, 0.8 W/cm^2^) for 300 s and the temperature was recorded at intervals of 10 s. PDA/Cur NFs (200 μg/mL) and PDA NTs (180 μg/mL) were exposed to laser irradiation (808 nm, 0.8 W/cm^2^) for 300 s and cooled at room temperature for 180 s. Then, the experiment was repeated five times to check the photothermal conversion stability, the temperature variation curve of PDA/Cur NFs (200 μg/mL) and PDA NTs (180 μg/mL) were recorded under NIR laser irradiation (808 nm, 0.8 W/cm^2^) for 300 s and cold at room temperature for 180 s. We further recorded the NIR images of the wound region after administration of PDA/Cur NFs (100 μL, 200 μg/mL) under NIR laser irradiation (808 nm, 0.8 W/cm^2^) for 300 s at intervals of 50 s using a near-infrared imager. The wound region after the administration of PDA/Cur NFs exposed to laser irradiation at 20 s intervals for a total of 300 s, and the temperature changes in the wounds were recorded with a near-infrared imager.

### In vitro antibacterial effects of PDA/Cur NFs

To assess the antibacterial activities of different concentrations of PDA/Cur NFs in vitro, MRSA suspensions (100 μL, 1 × 10^5^ CFU/mL) were added to the 96-well plates followed by incubation with a series of concentrations of Cur nanocrystals (100 μL; 5, 10, 15, and 20 μg/mL), PDA NTs (100 μL; 45, 90, 135, and 180 μg/mL), and PDA/Cur NFs (100 μL; 50, 100, 150, and 200 μg/mL). Unless stated otherwise, the concentrations of PDA NTs and Cur nanocrystals were in accordance with the contents of PDA/Cur NFs in this study. The LB solution was used as a control. The plates were irradiated with an NIR laser (808 nm, 0.8 W/cm^2^) for 300 s. Then, 10 μL of the bacterial suspension was transferred to LB agar and incubated for 16 h at 37 °C. The antibacterial activity was determined based on the number of CFUs using the plate-counting method.

To evaluate the antibacterial activities of different irradiation times in vitro*,* bacterial suspensions (100 μL, 1 × 10^5^ CFU/mL) were added in the 96-well plates followed by incubation with Cur nanocrystals, PDA NTs, and PDA/Cur NFs. The final concentration of PDA NT was 90 μg/mL, Cur nanocrystal was 10 μg/mL, and PDA/Cur NFs was 100 μg/mL. The irradiation times were 0, 2.5, 5, 7.5, and 10 min. Then, 10 μL of the bacterial inoculum was transferred to LB agar plates and bacterial colonies were counted after 16 h of incubation at 37 °C.

To evaluate the intrinsic antibacterial effects of the nanostructures, a series of concentrations of NFs (50, 100, 150, and 200 μg/mL), NTs (45, 90, 135, and 180 μg/mL), and Cur nanocrystals (5, 10, 15, and 20 μg/mL) were incubated with bacteria for 30 min without laser irradiation to detect the number of CFUs. In addition, different incubation times (10, 15, 30, and 60 min) with the NF dose of 100 μg/mL containing 90 μg/mL NTs and 10 μg/mL Cur were also investigated. All experimental groups were measured in triplicate.

### Confocal fluorescence assay

After incubation with PBS, PDA NTs (180 μg/mL), and PDA/Cur NFs (200 μg/mL) with or without NIR irradiation (808 nm, 0.8 W/cm^2^) for 300 s, and Cur nanocrystals (20 μg/mL) without NIR for 30 min, bacteria were stained with PI for 20 min, followed by SYTO™ 9 counterstaining for 10 min. Finally, the bacteria were visualized using a confocal fluorescence microscope (Leica, TCS SP8 STED, Germany).

### Morphology observation of bacteria

The incubation and irradiation procedures were performed using a confocal fluorescence assay. Two typical groups of bacterial dispersions (PBS control and PDA/Cur NFs + NIR) were harvested and fixed with 2.5% glutaraldehyde. Then, the bacteria were further dehydrated using a series of ethanol/water mixtures and dried by CO_2_ supercritical drying. Finally, the dried bacteria were observed by SEM (4800 II, Hitachi, Japan).

### Cytotoxicity

Cytotoxicity was measured by MTT assay and crystal violet staining in L929 and RAW264.7 cells. For MTT assay, cells (1 × 10^4^ cells/well) were seeded in 96-well plates, cultured for 12 h, and incubated with PBS, Cur nanocrystals, PDA NTs, and PDA/Cur NFs with or without NIR irradiation (808 nm, 0.8 W/cm^2^, and 300 s) for 24 h. The concentrations of Cur nanocrystals were 0.313–20 μg/mL, PDA NTs were 2.813–180 μg/mL, and PDA/Cur NFs were 3.125–200 μg/mL. Subsequently the cells were incubated with MTT solution (5 mg/mL) for 4 h and 100 μL DMSO was added, and the UV–Vis absorbance was measured at 490 nm using a microplate reader. Cell viability was normalized to PBS-treated cells and expressed as mean ± SD (n = 3). For crystal violet staining, cells (2 × 10^4^ cells/well) were seeded in 48-well plates, cultured for 12 h, and incubated with different concentrations (11.25–180 μg/mL) of PDA NTs for 24 h. PBS (0 μg/mL of PDA) was used as a control. Then the media was removed, and the cells were washed with PBS. The cells were fixed, stained with crystal violet, and photographed. As a semiquantitative measurement, 0.5% Triton X-100 solution was added overnight for extracting the crystal violet, and the absorbance was measured at 570 nm. Cell relative absorbance was normalized to PBS-treated cells and expressed as mean ± SD (n = 3).

### Wound scratch migration assay

L929 cells were seeded in 6-well plates (5 × 10^5^ cells/well) and allowed to form a confluent monolayer. Each well was gently scratched in a straight line using a 200 μL pipette tip. Plates were washed with PBS to remove floating cells and then treated with Cur nanocrystals, PDA NTs, and PDA/Cur NFs + NIR (808 nm, 0.8 W/cm^2^, and 300 s) containing 1% FBS and incubated at 37 °C for 24 h. The concentrations of Cur nanocrystals, PDA NTs, and PDA/Cur NFs were 10, 90, 100 μg/mL, respectively. The cells were stained with crystal violet and photographed at 0 and 24 h. Migration areas were quantitatively evaluated using Image-Pro Plus 6.0 software. Cell migration rates were calculated using the formula illustrated below:

$${\text{Cell migration rates }}\left( \% \right)\, = \,\left( {{\text{A}}_{{\text{t}}} - {\text{A}}_{0} } \right)/{\text{A}}_{0} \, \times \,{1}00\%$$

A_t_: The scratch area at 0 h.

A_0_: The scratch area at 24 h.

### Analysis of Mφ phenotype in vitro

RAW264.7 cells were seeded in 12-well plates (2 × 10^5^ cells/well) 12 h prior to the experiment. The cells were then treated with LPS (a final concentration of 10 μg/mL) and Cur nanocrystals, PDA NTs, and PDA/Cur NFs with or without NIR irradiation for 24 h. The concentrations of Cur nanocrystals, PDA NTs, and PDA/Cur NFs were 10, 90, 100 μg/mL, respectively. The cells were washed and stained with fluorescently labeled antibody APC-CD11b and, stained with fluorescently labeled antibodies PE-CD86, PE-CD80, and PE-CD206. Finally, all the samples were analyzed by flow cytometry.

### In vivo MRSA-infected diabetic wound healing

All animal experiments were conducted in accordance with the Animal Care and Use Committee of Yangzhou University. The wound healing effects of PDA/Cur NFs were evaluated in a diabetic MRSA-infected full-thickness cutaneous wound mouse model. In brief, 5-week-old male ICR mice were intraperitoneal injected with STZ (5 mg/kg) dissolved in citrate buffer (pH 4.5) once a day for five days. Mice with fasting blood glucose levels > 11.1 mmol/L for two continuous detections were defined as diabetic mice. Then, the diabetic mice were anesthetized and shaved on their backs. A round full-thickness cutaneous wound (8 mm × 8 mm) area was created on the back, after which 10 μL of MRSA solution (10^8^ CFU/mL) was introduced onto the wound. The mice were randomly divided into seven groups (n = 6): saline (100 μL) ± NIR, Cur nanocrystals (100 μL, 20 μg/mL), PDA NTs (100 μL, 180 μg/mL) ± NIR, and PDA/Cur NFs (100 μL, 200 μg/mL) ± NIR. The solutions were added on the infected wounds followed by NIR irradiation at 0.8 W/cm^2^ for 300 s. Wound sizes were measured using a digital caliper and photographed on 0, 3, 7, and 14 d. Wound healing rates were calculated according to the equation:

$${\text{Wound healing rate }}\left( \% \right)\, = \,({\text{W}}_{0} {-}{\text{W}}_{\vartriangle } )/{\text{W}}_{0} \, \times \,{1}00\%$$

W_△_: The wound area at certain day.

W_0_: The wound area at day 0.

For histological analysis, the wounds and major organs (heart, liver, spleen, lung, and kidney) were harvested on 3, 7, and 14 d, fixed in 4% formaldehyde, and embedded in paraffin for hematoxylin–eosin (H&E), Masson’s staining, and immunofluorescent staining of CD163, iNOS, VEGF, and Ki67 by Wuhan Servicebio Technology (Wuhan, China). The body weights of the mice were recorded every other day. To measure the number of bacteria in the infected wound tissues, the tissues were homogenized and diluted with sterile PBS and plated on LB agar for colony enumeration after 18 h of incubation at 37 °C.

### Hemolysis analysis

One milliliter of blood obtained from the mice was mixed with 3 mL PBS containing EDTA. Red blood cells were separated from the blood by centrifugation at 2000 rpm for 5 min. The cells were then washed thrice times with PBS and resuspended in PBS. Subsequently, 0.2 mL of diluted red blood cell suspension was added to 0.8 mL PBS (negative control), 0.8 mL deionized water (positive control), and 0.8 mL PDA/Cur NF dispersion at concentrations ranging from 6.25 to 1000 μg/mL. Finally, the samples were kept at room temperature for 4 h and centrifuged at 12,000 rpm for 10 min. The absorbance of the supernatant at 541 nm was measured using UV–Vis spectrophotometry. The calculation formula of hemolysis rate as follow:

$${\text{Hemolysis }}\left( \% \right)\, = \,({\text{A}}_{{{\text{NFs}}}} - {\text{A}}_{{{\text{PBS}}}} )/({\text{A}}_{{{\text{Water}}}} - {\text{A}}_{{{\text{PBS}}}} )\, \times \,{1}00\%$$

A: absorbance of the supernatant at 541 nm.

### Statistical analysis

Statistics were performed using GraphPad Prism 6 software. For the comparison of two groups a student’s *t* test was used. For more than two groups a two-way analysis-of-variance (ANOVA) was used followed by a Tukey post-hoc analysis to compare means between two groups. ^*^P < 0.05, ^**^P < 0.01, ^***^P < 0.001, ^****^P < 0.0001, ^#^*P* < 0.05, ^##^*P* < 0.01, ^###^*P* < 0.001, ^####^*P* < 0.0001, data represented as mean ± SD.

## Results and discussion

### Preparation and characterization of PDA/Cur NFs

**Fig. 1:**
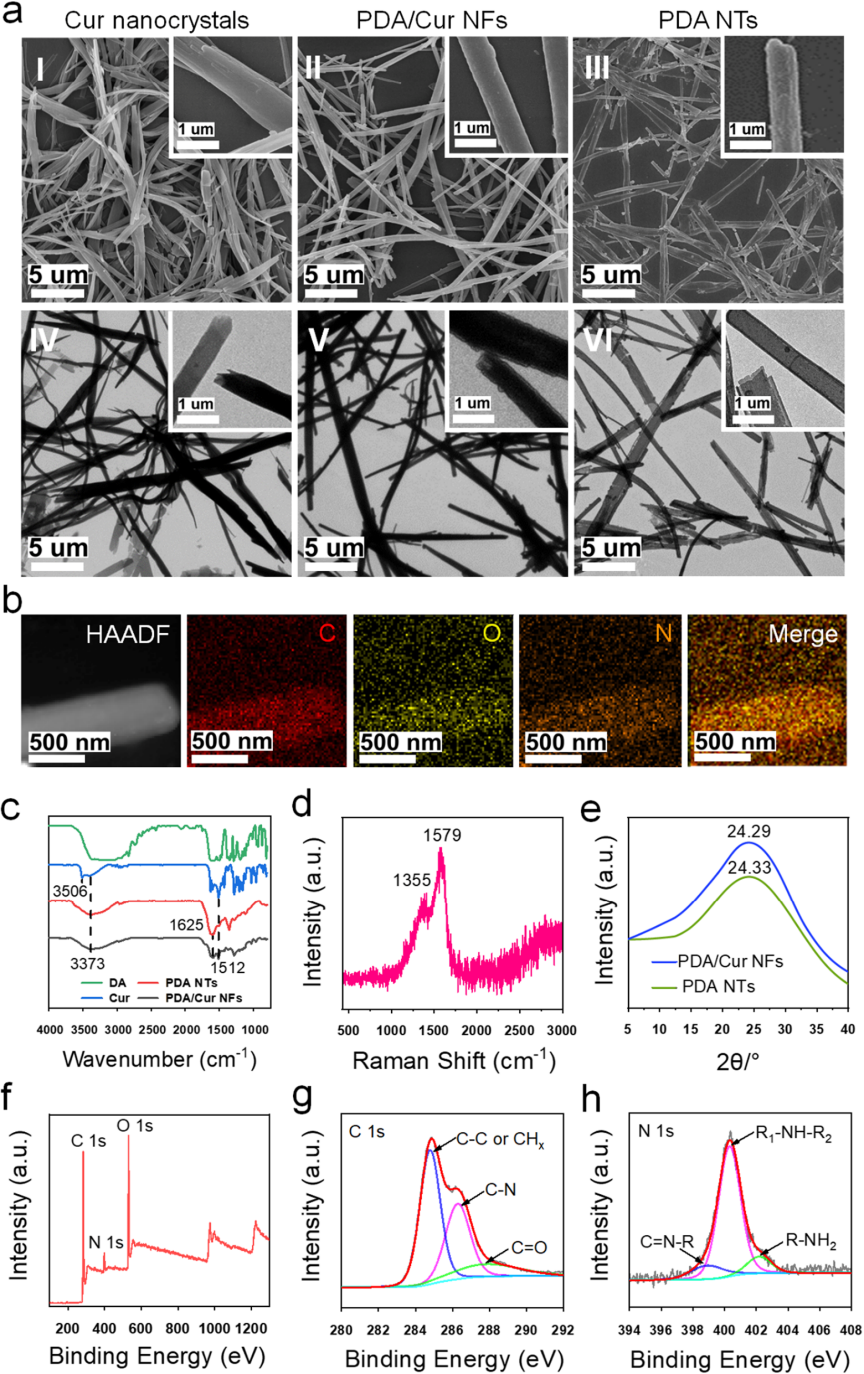
Characterization of Cur nanocrystals, PDA NTs, and PDA/Cur NFs. **a** Representative SEM images of Cur nanocrystals (I), PDA/Cur NFs (II), and PDA NTs (III). Representative TEM images of Cur nanocrystals (IV), PDA/Cur NFs (V), and PDA NTs (VI). **b** Elemental mapping images of PDA/Cur NFs. **c** FTIR spectrum of DA, Cur, PDA NTs and PDA/Cur NFs. **d** Raman spectrum of PDA/Cur NFs. **e** The XRD curve of PDA NTs and PDA/Cur NFs. **f**–**h** XPS of PDA/Cur NFs. **f** Full scan, **g** C 1 s high-resolution spectrum, **h** N 1 s high-resolution spectrum

The Cur nanocrystals were prepared by solvent exchange, the PDA/Cur NFs were produced via oxidative self-polymerization of DA, and PDA NTs were generated by dissolving Cur templates in NFs (Scheme 1a) [47]. The morphologies of the nanostructures were evaluated using SEM and TEM (Fig. 1a). SEM and TEM images of Cur nanocrystals showed fibrous shapes with a slightly curved, long (several tens of micrometers) length, and approximately 800 nm in diameter (Fig. 1a (I) and (IV)). The size of the PDA/Cur NFs was similar to that of the Cur nanocrystals, but more straight after the formation of the PDA shells (Fig. 1a (II) and (V)). The PDA NTs displayed hollow tubular structures after dissolving and removing the Cur core (Fig. 1a (III) and (VI)). Elemental mapping analysis revealed the uniform distribution of C, O, and N in the PDA/Cur NFs (Fig. 1b). C and O were present in both Cur and PDA, while N was derived from PDA, demonstrating the successful PDA coating.

FTIR spectroscopy was conducted to characterize the functional groups of PDA NTs and PDA/Cur NFs. The FTIR spectrum of the Cur peak emerged at 1512 cm^−1^ [48, 49], which could be ascribed to the mixed vibrations of ν(C = O), δ(CCC), δ(CC = O), aromatic ν(CC), and ν(CCH). Meanwhile, the PDA/Cur NFs exhibited a low intensity at 1512 cm^−1^ (Fig. 1c). Compared with PDA/Cur NFs and Cur crystals, PDA NTs showed a very weak peak at 1512 cm^−1^. This is because the Cur crystals have been removed after washing by ethanol. The peak at 3506 cm^−1^ corresponded to the phenolic OH-groups of Cur, which was not present in the spectrum of PDA NTs and PDA/Cur NFs. Compared with DA, the peak appearing at 1625 cm^−1^ of PDA NTs and PDA/Cur NFs can be attributed to the aromatic ring skeleton stretching vibration, while the broad peak at 2900–3700 cm^−1^ is due to the abundance of OH groups. The above results indicated that PDA/Cur NFs were successfully synthesized, which is in agreement with previous studies [50–53]. In addition to the peak at 1512 cm^−1^, other bands in the FTIR spectrum of Cur were almost absent in the spectrum of PDA/Cur NFs, possibly because the Cur loading was only 10%. This suggests that the interaction of Cur with the PDA coating lacks chemical bonding. On the other hand, the disappearance (possibly a red shift) of the band at 3506 cm^−1^ in PDA Cur NFs indicates H-bonding with OH groups. The band shape at 2900–3700 cm^−1^ which is characteristic of hydroxyl groups, also indicates a drastic change after PDA interacts with Cur. This change in shape is typical when OH groups form intramolecular hydrogen bonds [48, 54]. The above spectrum confirmed the PDA coating on the Cur nanocrystals.

In the PDA/Cur NFs, a broad and strong peak was observed at 1300–1600 cm^−1^ (Fig. 1d). The peaks centered at 1579 cm^−1^ could be assigned to ν (C=C) aromatic coupled with pyrrole ring stretching vibration or indole ring vibration from PDA coating [55] or Cur [56], and the peak at 1355 cm^−1^ was due to the aromatic ν(C-N) stretching mixed with indole ring stretching from the PDA coating. These results partly represent the structural features of the monomers composing PDA [57, 58]. The XRD spectrum of the PDA/Cur NFs exhibited a peak at 24.29, and the PDA NTs showed a peak at 24.33 (Fig. 1e), which is in accordance with previous research [47].

Figure 1f–h presented XPS characterization of PDA/Cur NFs, the N peak came from the N element of PDA shell, while the O and C peaks may have been derived from both PDA and Cur (Fig. 1f). C 1s high-resolution spectrum was fitted into three components: CHx or C=C, C–N, and C=O (Fig. 1g). The N 1s peak was fitted with three components: primary (R-NH_2_), secondary (R_1_-NH-R_2_) and tertiary/aromatic (C=N–R) amine functionalities (Fig. 1h). The high content of secondary amine indicates its formation followed cyclized self-polymerization path via 5, 6-dihydroxyindole [59].

As shown in UV–vis-NIR spectra (Additional file 1: Fig. S1), PDA Cur NFs showed similar absorption with PDA NTs, indicating that PDA coated Cur. Both PDA NTs and PDA/Cur NFs groups showed absorption in near-infrared areas, demonstrating the photothermal response capacity of PDA. Compared with PDA/Cur NFs without NIR irradiation, PDA/Cur NFs + NIR exhibited the typical absorption peak of Cur, suggesting that Cur is released after photothermal effects. The drug loading of Cur in PDA/Cur NFs was 10% determined by UV spectrophotometry. Consequently, the composite NFs were composed of PDA and Cur, and the content of PDA in NFs was 90%.

### Photothermal performance and drug release of PDA/Cur NFs

**Fig. 2:**
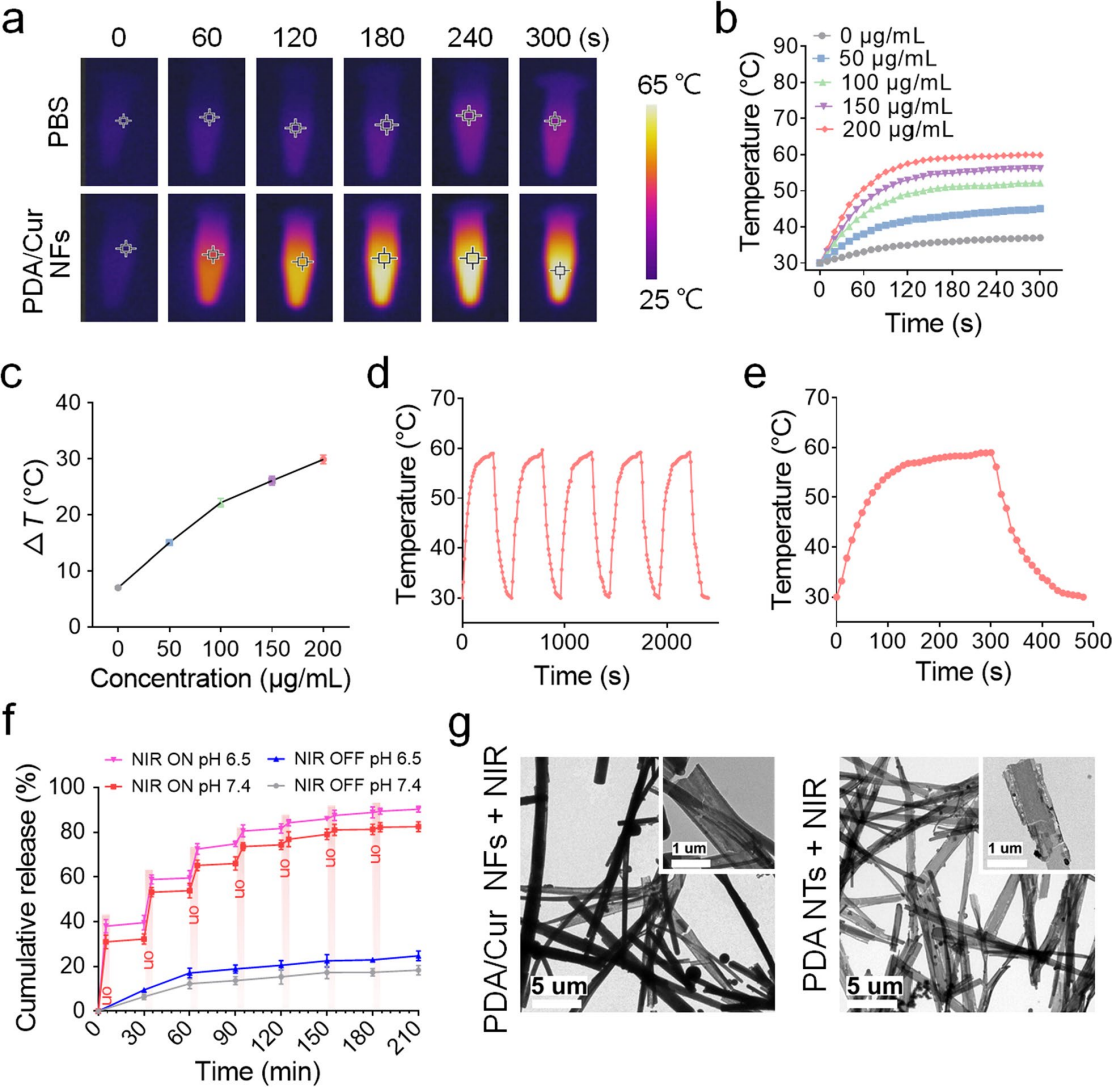
Photothermal effects and drug release of PDA/Cur NFs. **a** Thermal image of PDA/Cur NFs under NIR laser (808 nm, 0.8 W/cm^2^) for 300 s. **b** Temperature elevation curves of PDA/Cur NFs at different concentrations (0–200 μg/mL) upon NIR laser (808 nm, 0.8 W/cm^2^) for 300 s. **c** Plot of temperature change (Δ*T*) over a period of 300 s versus the concentration of PDA/Cur NFs. **d** Photothermal conversion ability of PDA/Cur NFs during 5 times of on/off NIR irradiation cycles. **e** Photothermal response of PDA/Cur NFs (200 μg/mL) for 300 s with NIR laser (808 nm, 0.8 W/cm^2^) and then the laser was shut off. **f** Cur release profile from PDA/Cur NFs with and without NIR irradiation in different pH condition (808 nm, 0.8 W/cm^2^). **g** The TEM images of PDA/Cur NFs and PDA NTs after exposure to laser irradiation (808 nm, 0.8 W/cm^2^) for 300 s. Data are presented as mean ± SD (n = 3)

Next, we evaluated the photothermal properties of PDA/Cur NFs. As shown in Fig. 2a, for PBS, there was no obvious temperature increase even after 300 s of NIR irradiation. In contrast, the photothermal response of PDA/Cur NFs (200 μg/mL) significantly increased under the same conditions. PDA/Cur NFs were dispersed in water and exposed to laser irradiation (808 nm, 0.8 W/cm^2^) for 300 s. The photothermal heating curves exhibited a concentration-dependent effect (Fig. 2b–c). It was found that NIR irradiation rapidly elevated the temperature to 55 °C within 2 min, and the maximum temperature reached 58 °C. After irradiation for 300 s, the temperature of the PDA/Cur NF aqueous solution notably increased at a concentration of 200 μg/mL. In contrast, the temperature of the pure water increased inconspicuously. To better assess whether such heating behavior could be repeatedly activated by NIR irradiation, the PDA/Cur NF solution underwent five consecutive on/off cycles of NIR exposure. For each cycle, the NFs quickly heated to approximately 58 °C when NIR was on, and immediately cooled to the initial temperature when NIR was switched off (Fig. 2d). The result in Fig. 2e explained the cure of a single temperature rise and fall under NIR exposure, up to 58 °C with 200 s when NIR was on and down to room temperature with 200 s when NIR was off. These results indicate that PDA/Cur NFs offer excellent photoresponsive heating behavior, which is beneficial for antibacterial therapy. We further investigated the photothermal effects of PDA NTs (Additional file 1: Fig. S2). The experimental procedure is the same as PDA/Cur NFs, and the photothermal performance of PDA NTs was consistent with that of PDA/Cur NFs, demonstrating the PDA NTs were good photothermal material for subsequent experiments.

The release of Cur from NFs is critical for exerting its regenerative effects; therefore, the release profile was investigated (Fig. 2f). The standard curve of Cur measured by HPLC was shown in Additional file 1: Fig. S3, and the R^2^ = 0.9997. The release rate of Cur from the PDA/Cur NFs was very slow, and the cumulative release amount of Cur only reached approximately 20% after 210 min without NIR irradiation at pH 7.4. However, a robust release of Cur was observed once the irradiation started, and the release became negligible when the irradiation was turned off. This cycle was repeated several times using laser irradiation. The reason why NIR irradiation could boost Cur release was further analyzed by TEM. Following laser exposure, the NFs became hollow and some crevices appeared, and the morphology was similar to that of the NTs (Fig. 2g). Therefore, the photothermal effects of NFs allowed on-demand release of inner Cur by disrupting the PDA shell coated on the crystals. Furthermore, the release of Cur could be accelerated at pH 6.5 with or without NIR irradiation (Fig. 2f), which proved the feasibility of application in the treatment of bacterial infection wounds [60].

### Antibacterial properties of PDA/Cur NFs

**Fig. 3:**
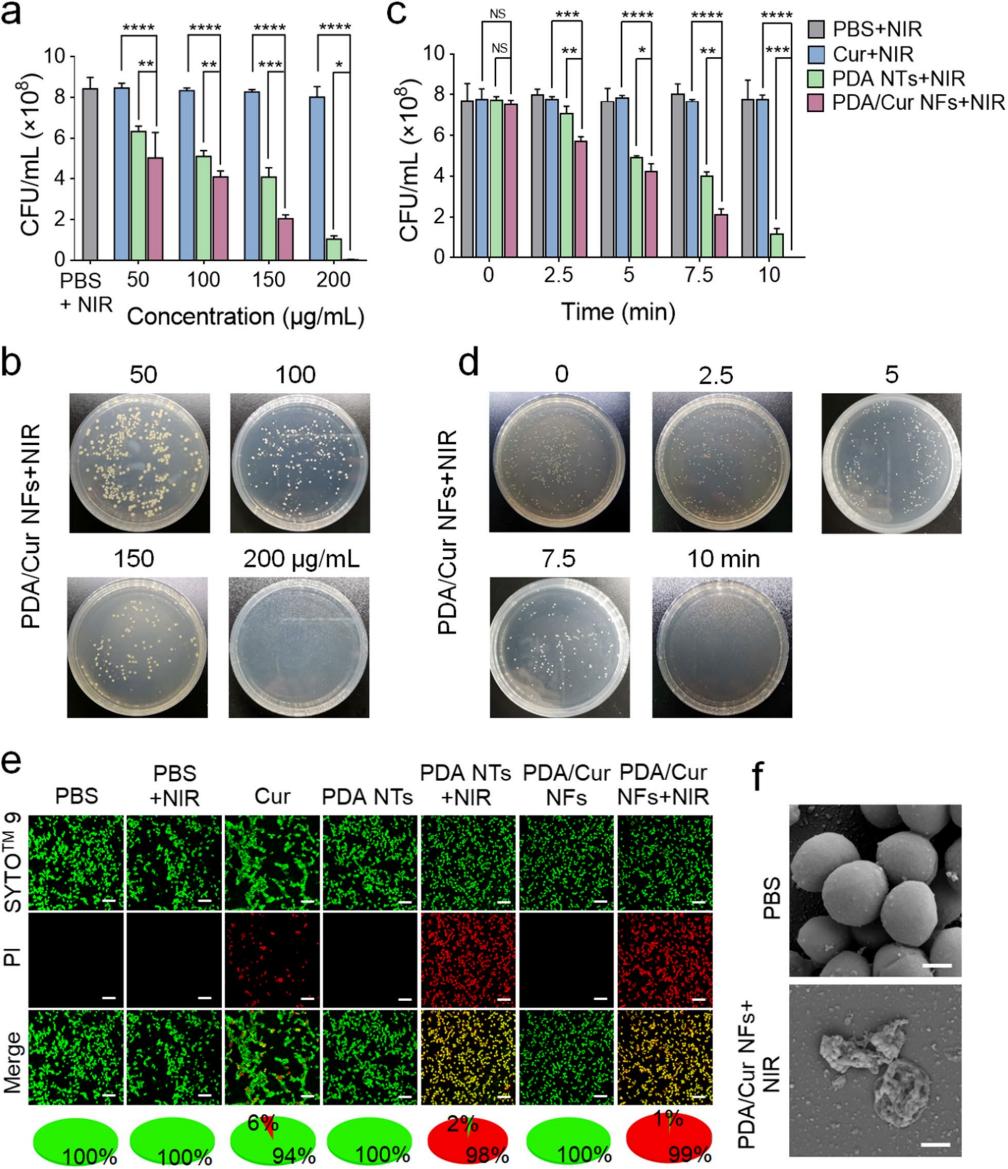
Antibacterial activity of PDA/Cur NFs. **a**–**b** Antibacterial effects and photographs of bacterial colonies of MRSA after being treated with of Cur nanocrystals, PDA NTs, and PDA/Cur NFs at different concentrations under NIR laser (808 nm, 0.8 W/cm^2^) for 300 s. **c**–**d** Antibacterial effects and photographs of bacterial colonies of MRSA after being treated with Cur nanocrystals (10 μg/mL), PDA NTs (90 μg/mL), and PDA/Cur NFs (100 μg/mL) under NIR laser (808 nm, 0.8 W/cm^2^) for different times. **e** Live/dead staining images of MRSA after different treatments (scale bar = 5 μm). **f** Representative SEM image of MRSA treated with PDA/Cur NFs (100 μg/mL) under NIR laser (808 nm, 0.8 W/cm^2^) for 300 s (scale bar = 200 nm). Data are presented as mean ± SD (n = 3). ^*^P < 0.05, ^**^P < 0.01, ^***^P < 0.001, ^****^P < 0.0001, vs the indicated groups

After confirming the photothermal performance of the PDA/Cur NFs, we focused our attention on their antibacterial potential in vitro. MRSA was used to test the antibacterial activity of the obtained NFs. Unless stated otherwise, the PDA/Cur NF concentrations were shown in the diagrams in this paper, which included the same contents of Cur and PDA with nanocrystals and NTs, respectively. Without laser irradiation, neither PDA NTs nor PDA/Cur NFs exhibited limited antibacterial activity up to a NF concentration of 200 μg/mL (Additional file 1: Fig. S4a–b) or an incubation time of 60 min (Additional file 1: Fig. S5a–b). However, after laser irradiation, the bactericidal capability of both NTs and NFs increased significantly in a dose and time-dependent manner (Fig. 3a–d, Additional file 1: Fig. S6a–b). At a concentration of 200 µg/mL and irradiation for 300 s, almost 100% and 87.7% of MRSA were killed by PDA/Cur NFs and PDA NTs, respectively (Fig. 3a–b, Additional file 1: Fig. S6a). Similarly, when the irradiation time was prolonged to 10 min, the antibacterial efficiencies of NFs and NTs were 100% and 84.8%, respectively, even at a concentration of 100 µg/mL (Fig. 3c–d, Additional file 1: Fig. S6b). These results indicate that the antibacterial activities of both NTs and NFs mainly originate from the photothermal effects. It is worth noting that PDA/Cur NFs displayed stronger antibacterial ability than PDA NTs (Fig. 3a–d, Additional file 1: Fig. S6a–b, *P* < 0.05), which was likely due to the bacteriostatic effects of Cur, such as downregulation of bacterial gene expression, interaction with DNA molecules, and self-oxidization to form intermediates [61, 62]. This speculation was also verified by the significant antibacterial effects of Cur nanocrystals as shown in Additional file 1: Fig. S4–S5 (*P* < 0.01).

To further confirm the photothermal antibacterial effects, the live and dead assay was performed [23, 63]. SYTO™ 9 is a membrane-permeant green fluorescent dye used to stain both live and dead cells. PI is a red fluorescent dye that stains dead bacteria with damaged cell membranes [64, 65]. As shown in Fig. 3e, the bacteria in the PBS group showed almost no red fluorescence even after irradiation, indicating that the bacteria remained alive. The Cur crystal-treated group (20 µg/mL, 30 min) displayed a limited antibacterial effect, with 6% bacteria dead. However, nearly all of the bacteria showed red staining after being treated with PDA NTs and PDA/Cur NFs upon NIR irradiation, suggesting that the bacteria were destroyed. SEM was used to observe the morphologies of MRSA treated with (Fig. 3f). The bacteria in the PBS group presented their typical spherical shapes, while MRSA suffered from pronounced membrane damage and bacterial morphology destruction in the presence of PDA/Cur NFs plus NIR irradiation. Consequently, these results demonstrate that PDA/Cur NFs possess promising photothermal antibacterial abilities with the assistance of NIR irradiation.

### Cell scratch healing and Mφ polarization of PDA/Cur NFs

The second stage of sequential therapy should be the proliferation phase, and M2 Mφs are involved in terminating inflammation and initiating wound repair [66]. Herein, we determined the in vitro wound-healing activity of NFs. First, we evaluated the cytotoxicity of NFs in L929 and RAW264.7 cells. As shown in Additional file 1: Fig. S7a, none of the treatments of Cur nanocrystals, PDA NTs, and PDA/Cur NFs ± NIR induced any significant decrease in L929 cell viability even at a high concentration of 200 μg/mL after 24 h of incubation. In RAW264.7 cells, we observed no significant cytotoxicity associated with NF or NT treatments at a concentration of 100 μg/mL (> 90% cell viability, Additional file 1: Fig. S8a). The changes in the number of L929 cells and RAW264.7 cells were consistent with the results of the MTT assay. (Additional file 1: Fig. S7b–c, S8b–c). These cytotoxicity results can be used as guidance for subsequent cell experiments.

**Fig. 4:**
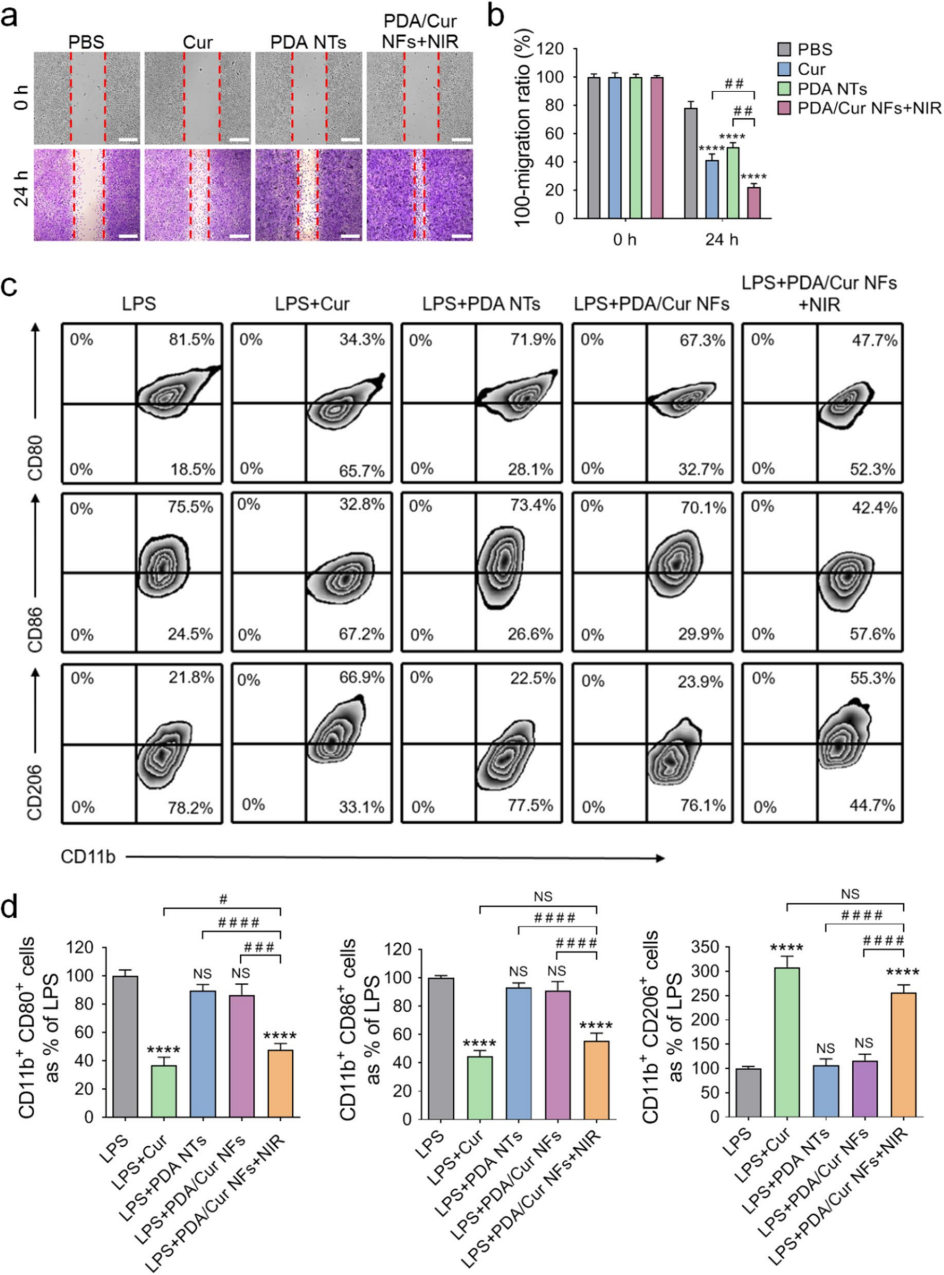
Cell scratch healing and Mφ polarization of PDA/Cur NFs. **a** Images for cell wound scratch assay of L929 cells (scale bar = 100 μm). **b** The quantitative analysis of the scratch area. **c** Representative flow cytometric analysis images and **d** relative quantification of CD11b^+^CD80^+^, CD11b^+^CD86^+^, and CD11b^+^CD206^+^ cells after different treatments. Data are presented as mean ± SD (n = 3). ^****^P < 0.0001, vs PBS or LPS groups. ^#^*P* < 0.05, ^##^*P* < 0.01, ^###^*P* < 0.001, ^####^*P* < 0.0001, vs the indicated groups. NS, no significant difference

The scratch assay was used to further determine the effect of biomedical materials on cell viability and migration [67–70]. To verify the effect of PDA/Cur NFs in accelerating dermal tissue restoration, we chose L929 cells for the in vitro scratch assay. Following the different cell scratch treatments, the extent of migration was significantly different (Fig. 4a). Cur is well known as a wound healing agent [42, 71], which showed faster scratch healing, resulting in an approximately 2.7-fold faster migration rate than the PBS group (Fig. 4b). PDA NTs also showed a high scratch closure rate similar to that of the Cur group, which could be due to the enhanced cell adhesion by PDA, which is beneficial to cell proliferation (Fig. 4a, b). After treatment with PDA/Cur NFs under laser irradiation, the cells exhibited the strongest migration capability (Fig. 4a). Quantitative analysis was also in agreement with the migration observation, where the group treated with PDA/Cur NFs plus laser irradiation showed the highest migration rate (*P* < 0.01, vs. Cur and PDA NTs, Fig. 4b).

Dynamic changes in the Mφs phenotype play an important role in the transition from the inflammatory phase to the proliferation phase during the process of wound healing [67]. Therefore, we estimated the population of M1 (CD11b^+^CD80^+^ and CD11b^+^CD86^+^) and M2 (CD11b^+^CD206^+^) using flow cytometry. After treating Mφs with LPS, approximately 80% of M1 Mφs (CD11b^+^CD80^+^, 81.5%; CD11b^+^CD86^+^, 75.5%) were detected (Fig. 4c–d). Treatment with PDA NTs and PDA/Cur NFs in the presence of LPS showed no significant differences from the LPS group (NS). However, PDA/Cur NFs + NIR treatment resulted in a large reduction in M1 Mφs (CD11b^+^CD80^+^, 47.7%; CD11b^+^CD86^+^, 42.4%) and an increase in M2 phenotype (CD11b^+^CD206^+^, 55.3%), which was comparable to that of the Cur nanocrystal group, suggesting that Mφs were reprogrammed from the M1 phenotype to the M2 phenotype by the release of Cur.

### MRSA-infected diabetic wounds healing using PDA/Cur NF treatment

**Fig. 5:**
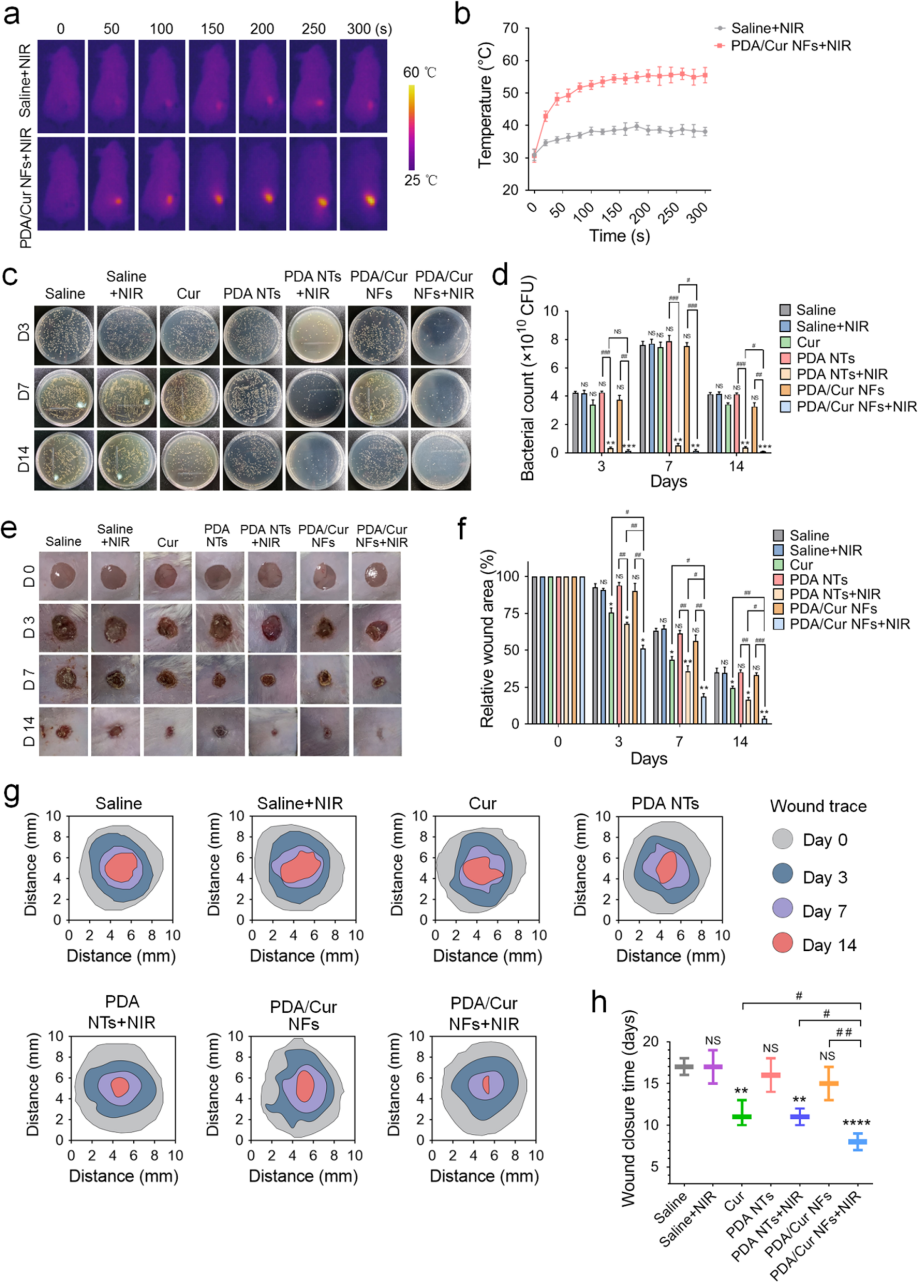
MRSA-infected diabetic wounds healing using PDA/Cur NFs treatment. **a**–**b** In vivo photothermal images and wound temperature increase curve of the MRSA-infected diabetic wounds treated with PDA/Cur NFs under NIR laser (808 nm, 0.8 W/cm^2^) for 300 s. **c**–**d** Photographs and quantitative analysis of the bacterial colony of the infected wounds treated with different treatment at day 3, 7, and 14. **e** The representative images of the wound healing process of mice with different treatments. **f** Wound closure rates at different time points of each group. **g** Traces of wound-bed closure during 14 days for each treatment. **h** Wound closure time for each treatment. Data are presented as mean ± SD (n = 6). ^*^P < 0.05, ^**^P < 0.01, ^***^P < 0.001, ^****^P < 0.0001, vs saline group on the same day. ^#^*P* < 0.05, ^##^*P* < 0.01, ^###^*P* < 0.001, vs the indicated groups. NS, not signifcant vs saline group on the same day

Before testing the in vivo antibacterial activity, we confirmed that the PDA/Cur NFs in the wounds were sufficient to achieve substantial heating upon NIR irradiation. Diabetic mice bearing MRSA-infected wounds were dropped with PDA/Cur NFs (100 μL, 200 μg/mL) following exposure to an 808 nm laser (0.8 W/cm^2^, 300 s). As monitored by photothermography, the local wound temperature of mice treated with PDA/Cur NFs quickly increased to 52 °C within 100 s and followed up to 55 °C after irradiation for 200 s (Fig. 5a–b), which was deemed high enough to kill bacteria [23, 63]. However, under the same irradiation conditions, no obvious increase in temperature could be detected in the saline group. Therefore, the in vivo photothermal effect analysis further verified that PDA/Cur NFs could serve as a promising nanomaterial for PTT.

After confirming the in vivo photothermal effect, we evaluated the antibacterial activity of the NFs in diabetic wounds using a standard plate counting method (Additional file 1: Fig. S9). To demonstrate the effects of the components in NFs, we divided the mice into seven groups: saline, saline ± NIR, Cur nanocrystals, PDA NTs, PDA NTs ± NIR, PDA/Cur NFs, and PDA/Cur NFs ± NIR. As shown in Fig. 5c–d, the PDA/Cur NFs + NIR and PDA NT + NIR groups exhibited much fewer bacterial colonies than the saline control (*P* < 0.01) and the relevant groups (*P* < 0.01) without laser exposure, revealing that PDA as a photothermal agent possessed excellent photothermal antibacterial ability in vivo. As expected, the PDA/Cur NFs + NIR group showed the most outstanding antibacterial ability with almost 100% reduction in CFU counting, which was superior to that of the PDA NTs + NIR group. This phenomenon was similar to that of the in vitro antibacterial results shown in Fig. 3a–d.

Subsequently, the wound healing effect of the PDA/Cur NFs was evaluated. As anticipated, the wounds of mice treated with Cur nanocrystals (day 3, *P* < 0.05; day 7, *P* < 0.05; day 14, *P* < 0.05), PDA NTs + NIR (Day 3, *P* < 0.05; Day 7, *P* < 0.01; Day 14, *P* < 0.05), and PDA/Cur NFs + NIR (day 3, *P* < 0.05; day 7, *P* < 0.01; day 14, *P* < 0.01) showed significantly accelerated wound closure compared to the saline control on the same day (Fig. 5e–f). More precisely, the PDA/Cur NFs + NIR treatment led to 96.3% wound closure on day 14, which was significantly higher than that of the Cur nanocrystals (75.3%, *P* < 0.01) and PDA NTs + NIR (83.3%, *P* < 0.05). The fastest wound recovery of PDA/Cur NFs + NIR could be attributed to the combined advantages of PDA and Cur. In contrast to PDA/Cur NFs + NIR, the PDA/Cur NFs showed negligible therapeutic effect due to the Cur coated by PDA shell as well as the absence of a photothermal effect. The saline + NIR group showed no observed wound healing effect compared to the saline control group, indicating that laser treatment alone is far from sufficient to promote wound tissue regeneration. Moreover, Fig. 5g showed the traces of wound bed closure during 14 days for each treatment, which was consistent with the wound healing results in Fig. 5e–f. The wound closures of different treatments are shown in Fig. 5h, and the wound in the PDA/Cur NF group was closed at day 8 compared with the wound in the saline group at day 17.

### Histomorphological analysis of wound regeneration

**Fig. 6:**
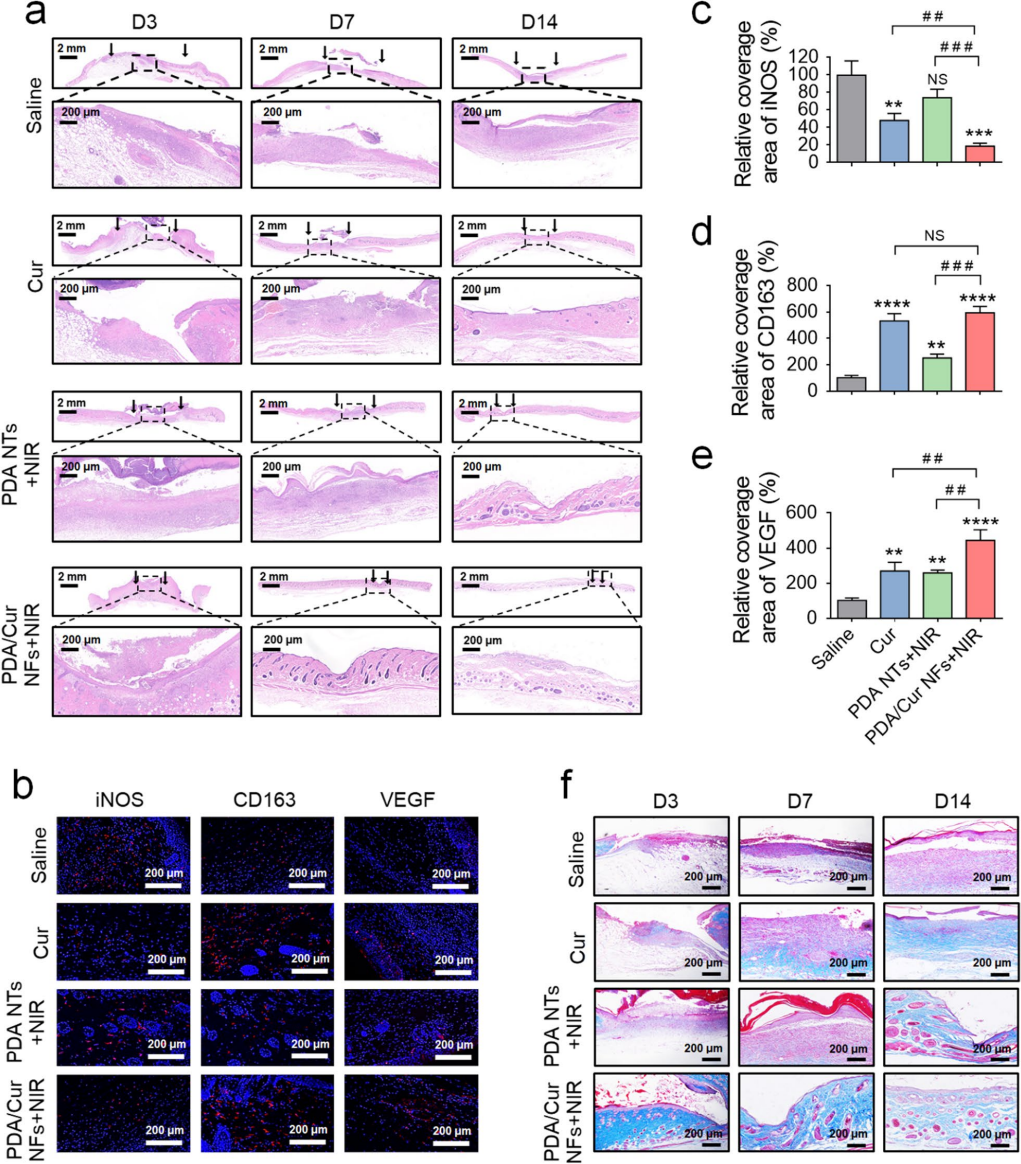
Histomorphological analysis of wound regeneration. **a** H&E staining of wounds treated with saline, Cur, PDA NTs + NIR, and PDA/Cur NFs + NIR on days 3, 7, and 14. Black arrows indicated the wound boundaries. **b** Representative immunofluorescence images showing iNOS, CD163, and VEGF for different groups. **c**–**e** The relative quantification of iNOS, CD163, and VEGF after different treatments. **f** Masson staining of wounds treated with saline, Cur, PDA NTs + NIR, and PDA/Cur NFs + NIR on days 3, 7, and 14. Data are presented as mean ± SD (n = 3). ^**^P < 0.01, ^***^P < 0.001, ^****^P < 0.0001, vs saline group. ^##^*P* < 0.01, ^###^*P* < 0.001, vs the indicated groups. NS, no significant difference

The wound healing in different groups on days 3, 7, and 14 was further evaluated by H&E staining. Black arrows indicate the wound boundaries, and the results are presented in Fig. 6a and Additional file 1: Fig. S10. On day 3, tissue necrosis and inflammation were observed in the wound sections, which were mainly induced by bacterial infection and the migration of inflammatory cells [72]. On day 7, the PDA/Cur NFs + NIR, PDA NT + NIR, and Cur groups exhibited varying degrees of healing, with PDA/Cur NFs + NIR emerging the best repair effect. On day 14, the formation of the epidermis layers was observed in all groups, although the thickness was different. Compared with other groups, the wound in the PDA/Cur NFs + NIR group was nearly closed, and more hair follicles regenerated. Although the healing effects of Cur nanocrystals and PDA NTs + NIR were not as perfect as those of PDA/Cur NFs + NIR, the inflammatory areas were significantly reduced and the wound surface was tidy compared with that of saline, saline + NIR, PDA NTs, and PDA/Cur NFs. This is because Cur possesses the ability to fight inflammation and promote tissue repair [71], while PDA can reduce MRSA infection through photothermal activity. Overall, the PDA/Cur NFs combined the characteristics of both Cur and PDA, which achieved the best healing effects.

iNOS is a marker of M1 Mφs, which plays a promoting role in the occurrence and development of inflammation [73, 74]. CD163 is the M2 marker that is observed during the recovery stage of inflammation or when inflammation is under control [38]. Immunofluorescence staining showed that PDA/Cur NFs + NIR could up-regulate the M1-type index of Mφs and downregulate the M2-type index, which was similar to Cur crystal treatment and consistent with the in vitro experiments (Fig. 4c–d), indicating that PDA/Cur NFs under laser exposure possess the capability to promote inflammatory-proliferation phase transition in wound healing in vivo by reprogramming Mφs (Fig. 6b–d).

M2 Mφs are known to promote collagen production, angiogenesis, and cell proliferation, thus playing critical roles in wound repair [75]. Masson staining can distinguish collagen fibers and muscle fibers in new tissues of the wound, while collagen fiber is the raw material for the formation of new tissues [76, 77]. Therefore, masson staining was used to reflect the situation of the wound regeneration. As shown in Fig. 6f and Additional File 1: Fig. S11, more collagen (blue color) formation and deposition was observed in the regenerated tissues of the PDA/Cur NFs + NIR group compared to other groups on days 3 and 7. The Cur nanocrystal group also displayed collagen deposition to a certain extent on day 7, which was due to the promotion of tissue repair by Cur [42]. Most of the groups gained varying degrees of collagen deposition in connective tissues on day 14, which resulted from the self-repair ability of the body. However, the PDA/Cur NFs + NIR treatment was approximately restored normally by this time. To further determine the revascularization mediated by PDA/Cur NFs in the wound healing process, VEGF staining was further tested on the subcutaneous tissue on day 14. In this study, all treatments significantly increased the number of VEGF-positive cells, especially for PDA/Cur NFs + NIR (Fig. 6b and e). Immunofluorescence staining of Ki67 was performed to investigate cell proliferation [78]. As shown in Additional file 1: Fig. S12a–b, extended areas with Ki67-positive cells were observed in the PDA/Cur NFs + NIR group, suggesting substantially increase in cell proliferation in the wound tissues, thereby expediting the formation of granulation tissue and enhancing collagen deposition [78] (Fig. 6f).

In conclusion, PDA/Cur NFs + NIR exhibited remarkable therapeutic efficacy in healing bacteria-infected diabetic wounds by promoting M2 polarization, depositing collagen, stimulating angiogenesis, accelerating cell proliferation, and finally reconstructing impaired skin.

### Biocompatibility evaluation of PDA/Cur NFs in vivo

**Fig. 7:**
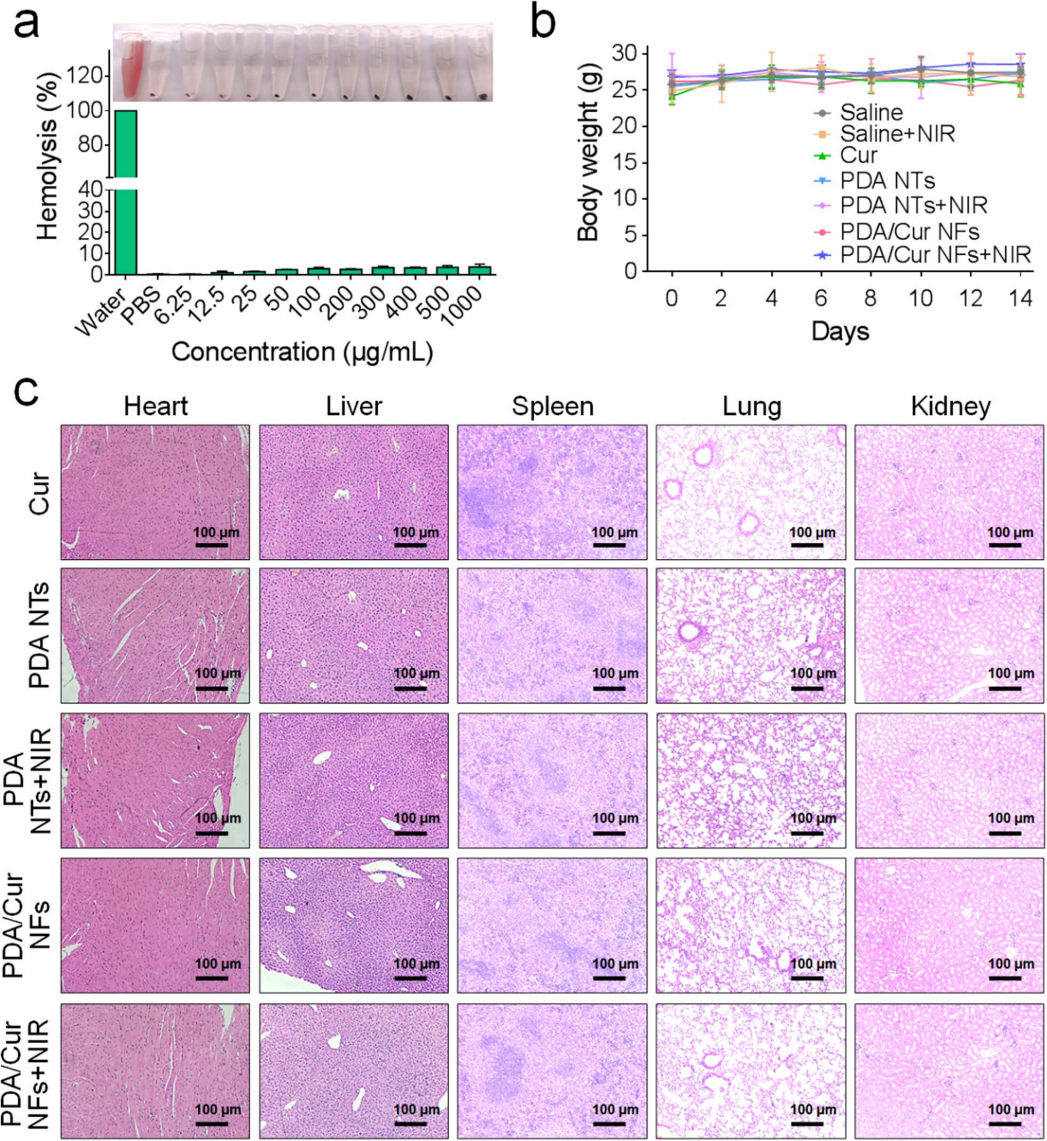
Safety assessment of PDA/Cur NFs. **a** Hemolysis assessment of PDA/Cur NFs at different concentrations (n = 3). **b** Body weight during 14 days after different treatments (n = 6). **c** H&E stained images of heart, liver, spleen, lung, and kidney at day 14 after different treatments. Data are presented as mean ± SD

Lastly, we evaluated the biocompatibility by detecting hemolysis, weight changes and H&E staining of major organs of mice treated with PDA/Cur NFs. The water-treated and PBS-treated groups were positive and negative controls, and their hemolysis rates were 100% and 0, respectively. When incubated with PDA/Cur NFs, the hemolysis rate was only 5% with a concentration up to 1000 μg/mL, suggesting that PDA/Cur NFs had excellent biocompatibility (Fig. 7a). We further measured the bodyweight of the mice during the entire period of treatment. Compared with the saline group, no significant change was observed among any of the treated groups (Fig. 7b). Pathological examination of main organs is an important means to evaluate safety. Thus major organs (heart, liver, spleen, lung, and kidney) were collected and histologically analyzed. H&E staining demonstrated no detectable toxicity in any organ (Fig. 7c). Therefore, these results support the safety of PDA/Cur NFs.

## Conclusion

In our study, we reported a nanofiber with the ability of photothermal antibacterial and Mφ polarization. It was validated that PDA/Cur NFs achieved responsive release of Cur which could switch Mφ from the M1 to M2 phenotype and promote cell migration in vitro. In an MRSA-infected diabetic mouse model, PDA/Cur NFs helped to kill bacteria and promote the transition from the inflammatory phase to proliferation by polarizing M2 Mφs, and subsequent deposition of collagen, stimulating angiogenesis, and facilitating cell proliferation to accelerate wound healing. Overall, this study provides a promising sequential therapy strategy depending on the initial killing of bacteria and subsequent M2 Mφ polarization to improve the outcomes of bacteria-infected diabetic wounds.

## Supplementary Information

**Additional file 1**: **Fig. S1.** UV–Vis-NIR spectra of Cur, PDA NTs, PDA/Cur NFs, and PDA/Cur NFs + NIR. **Fig. S2.** (a) Thermal image of PDA NTs under NIR laser (808 nm, 0.8 W/cm^2^) for 300 s. (b) Temperature elevation curves of PDA NTs at different concentrations (0–180 μg/mL) upon NIR laser (808 nm, 0.8 W/cm^2^) for 300 s. (c) Plot of temperature change (*ΔT*) over a period of 300 s versus the concentration of PDA NTs. (d) Photothermal conversion ability of PDA NTs during 5 times of on/off NIR irradiation cycles. (e) Photothermal response of PDA NTs (180 μg/mL) for 300 s with NIR laser (808 nm, 0.8 W/cm^2^) and then the laser was shut off. **Fig. S3.** Standard curve of Cur measured by HPLC. **Fig. S4.** (a) Antibacterial effects of Cur nanocrystals, PDA NTs, and PDA/Cur NFs at different concentrations for 30 min incubation. (b) Photographs of bacterial colonies of MRSA after being treated with Cur nanocrystals, PDA NTs, and PDA/Cur NFs for 30 min at different concentrations. **Fig. S5.** (a) Antibacterial effects of Cur nanocrystals (10 μg/mL), PDA NTs (90 μg/mL), and PDA/Cur NFs (100 μg/mL) for different incubation times. (b) Photographs of bacterial colonies of MRSA after being treated with Cur nanocrystals (10 μg/mL), PDA NTs (90 μg/mL), and PDA/Cur NFs (100 μg/mL) for different incubation times. **Fig. S6.** (a) Photographs of bacterial colonies of MRSA after being incubated with Cur nanocrystals and PDA NTs at different concentrations under NIR laser (808 nm, 0.8 W/cm^2^, 300 s). (b) Photographs of bacterial colonies of MRSA after being treated with Cur nanocrystals (10 μg/mL) and PDA NTs (90 μg/mL) under NIR laser (808 nm, 0.8 W/cm^2^) for different times. **Fig. S7.** (a) The cell viability of L929 cell after being treated with Cur nanocrystals, PDA NTs, PDA/Cur NFs, and PDA/Cur NFs + NIR at 24 h post-incubation with different concentrations. (b–c) The crystal violet staining and relative absorbance of L929 cell after being treated with PDA NTs at 24 h post-incubation with different concentrations (scale bar = 50 μm). **Fig. S8**. (a) The cell viability of RAW264.7 cell after being treated with Cur nanocrystals, PDA NTs, PDA/Cur NFs, and PDA/Cur NFs + NIR at 24 h post-incubation with different concentrations. (b–c) The crystal violet staining and relative absorbance of RAW264.7 cell after being treated with PDA NTs at 24 h post-incubation with different concentrations (scale bar = 50 μm). **Fig. S9.** A schematic illustration showing the in vivo infected diabetic wounds at different time intervals under treatment. **Fig. S10.** H&E staining of wounds treated with saline + NIR, PDA NTs, and PDA/Cur NFs on days 3, 7, and 14. Black arrows indicated the wound boundaries. **Fig. S11.** Masson staining of wounds treated with saline + NIR, PDA NTs, and PDA/Cur NFs on days 3, 7, and 14. **Fig. S12.** (a) Representative immunofluorescence images showing Ki67 for different groups. (b) The relative quantification of Ki67 after different treatments. Data are presented as mean ± SD (n = 3). ^**^P < 0.01, ^***^P < 0.001, ^****^P < 0.0001, vs saline group. ^##^*P* < 0.01, ^###^*P* < 0.001, vs the indicated groups.

## Data Availability

The datasets used and analyzed during the current study are available from the corresponding author on reasonable request.

## Abbreviations

MRSA: Methicillin-resistant *Staphylococcus aureus*
PTT: Photothermal therapy
NPs: Nanoparticles
DA: Dopamine
PDA: Polydopamine
Mφs: Macrophages
Cur: Curcumin
NFs: Nanofibers
NTs: Nanotubes
Tris-HCl: Tris hydrochloride
LPS: Lipopolysaccharide
MTT: 3-[4,5-Diamethylthiazol-2-yl]2,5-diphenyltetrazoliumbromide
STZ: Streptozotocin
DMEM: Dulbecco’s Modified Eagle’s Medium
FBS: Fetal bovine serum
DAPI: 4, 6-Diamidino-2-phenylindole
PI: Propidium iodide
