# Supplementary material for: Nanofiber-mediated sequential photothermal antibacteria and macrophage polarization for healing MRSA-infected diabetic wounds
Source: J Nanobiotechnology. 2021 Dec 5;19:404. doi: 10.1186/s12951-021-01152-4 (PMC8647563; doi:10.1186/s12951-021-01152-4)
Supplement: Supplementary file 1 — Additional file 1: Fig. S1. UV–Vis-NIR spectra of Cur, PDA NTs, PDA/Cur NFs, and PDA/Cur NFs + NIR. Fig. S2. (a) Thermal image of PDA NTs under NIR laser (808 nm, 0.8 W/cm2) for 300 s. (b) Temperature elevation curves of PDA NTs at different concentrations (0–180 μg/mL) upon NIR laser (808 nm, 0.8 W/cm2) for 300 s. (c) Plot of temperature change (ΔT) over a period of 300 s versus the concentration of PDA NTs. (d) Photothermal conversion ability of PDA NTs during 5 times of on/off NIR irradiation cycles. (e) Photothermal response of PDA NTs (180 μg/mL) for 300 s with NIR laser (808 nm, 0.8 W/cm2) and then the laser was shut off. Fig. S3. Standard curve of Cur measured by HPLC. Fig. S4. (a) Antibacterial effects of Cur nanocrystals, PDA NTs, and PDA/Cur NFs at different concentrations for 30 min incubation. (b) Photographs of bacterial colonies of MRSA after being treated with Cur nanocrystals, PDA NTs, and PDA/Cur NFs for 30 min at different concentrations. Fig. S5. (a) Antibacterial effects of Cur nanocrystals (10 μg/mL), PDA NTs (90 μg/mL), and PDA/Cur NFs (100 μg/mL) for different incubation times. (b) Photographs of bacterial colonies of MRSA after being treated with Cur nanocrystals (10 μg/mL), PDA NTs (90 μg/mL), and PDA/Cur NFs (100 μg/mL) for different incubation times. Fig. S6. (a) Photographs of bacterial colonies of MRSA after being incubated with Cur nanocrystals and PDA NTs at different concentrations under NIR laser (808 nm, 0.8 W/cm2, 300 s). (b) Photographs of bacterial colonies of MRSA after being treated with Cur nanocrystals (10 μg/mL) and PDA NTs (90 μg/mL) under NIR laser (808 nm, 0.8 W/cm2) for different times. Fig. S7. (a) The cell viability of L929 cell after being treated with Cur nanocrystals, PDA NTs, PDA/Cur NFs, and PDA/Cur NFs + NIR at 24 h post-incubation with different concentrations. (b–c) The crystal violet staining and relative absorbance of L929 cell after being treated with PDA NTs at 24 h post-incubation with different concentrat [file 12951_2021_1152_MOESM1_ESM.docx]

*Additional file 1*

**Nanofiber-mediated sequential photothermal antibacteria and macrophage** **polarization for healing MRSA-infected diabetic wounds**

Zhou Xu1 †, Bin Deng^2^ †, Xuewen Wang1 †, Jie Yu^3^, Zhuobin Xu^4^, Penggang Liu1, Caihong Liu1, Yuan Cai1, Fei Wang1, Rongling Zong1, Zhiling Chen1, Hua Xing1, Gang Chen1,5 *

1*Institute of Comparative Medicine, College of Veterinary Medicine, Jiangsu Co-Innovation Center for Prevention and Control of Important Animal Infectious Diseases and Zoonoses, Joint International Research Laboratory of Agriculture and Agri-Product Safety, The Ministry of Education of China, Yangzhou University, Yangzhou 225009, China*

^2^*Department of Gastroenterology, Affiliated Hospital, Yangzhou University, Yangzhou 225009, China*

^3^*Department of Traditional Chinese Medicine, Affiliated Hospital, Yangzhou University, Yangzhou 225009, China*

^4^*Institute of Translational Medicine, Medical College, Yangzhou University, Yangzhou 225001, China*

^5^*School of Basic Medical Sciences, Xuzhou Medical University, Xuzhou, 221004, China*

*Correspondence: gang_chen2015@163.com (G. Chen)

†Zhou Xu, Bin Deng, and Xuewen Wang contributed equally to this work.

**Fig. S1** UV-Vis-NIR spectra of Cur, PDA NTs, PDA/Cur NFs and PDA/Cur NFs+NIR.


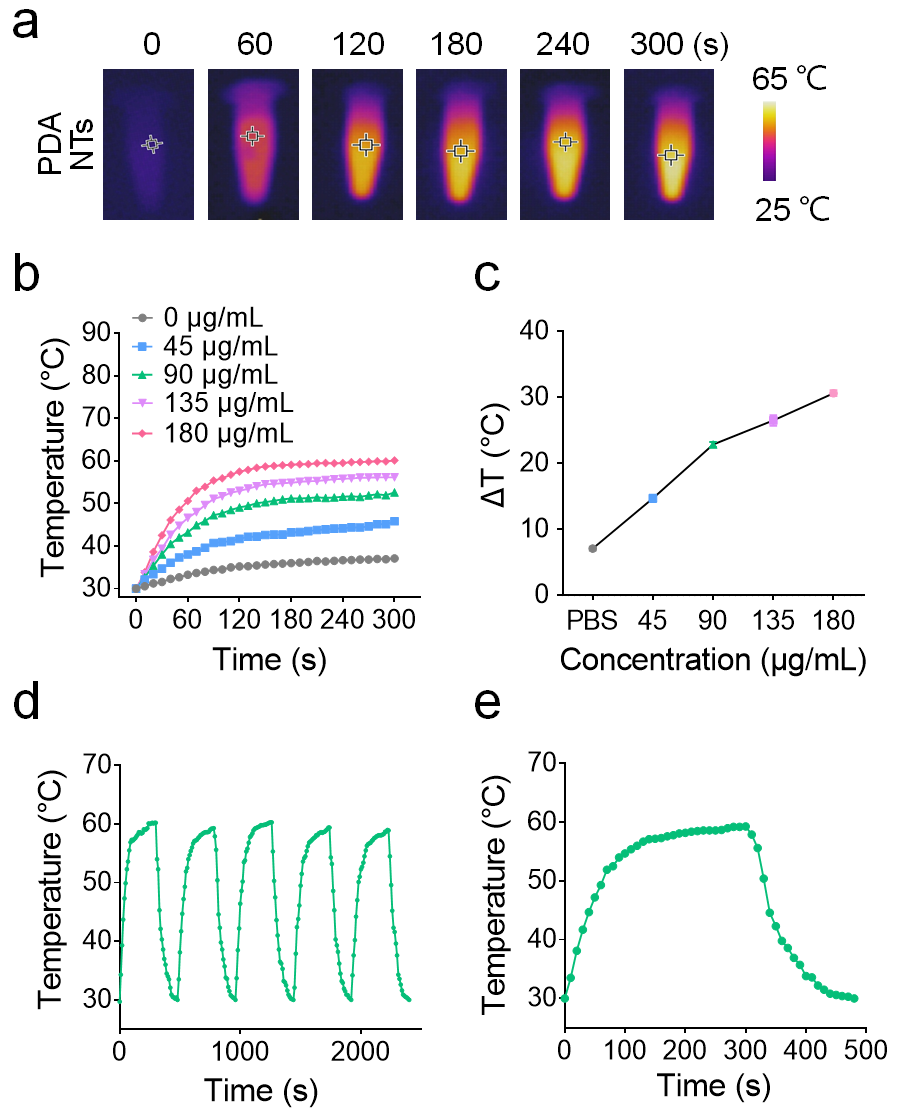


**Fig. S2 Photothermal effects of PDA NTs.** (a) Thermal image of PDA NTs under NIR laser (808 nm, 0.8 W/cm^2^) for 300 s. (b) Temperature elevation curves of PDA NTs at different concentrations (0~180 μg/mL) upon NIR laser (808 nm, 0.8 W/cm^2^) for 300 s. (c) Plot of temperature change (Δ*T*) over a period of 300 s versus the concentration of PDA NTs. (d) Photothermal conversion ability of PDA NTs during 5 times of on/off NIR irradiation cycles. (e) Photothermal response of PDA NTs (180 μg/mL) for 300 s with NIR laser (808 nm, 0.8 W/cm^2^) and then the laser was shut off.

**Fig. S3** Standard curve of Cur measured by HPLC.


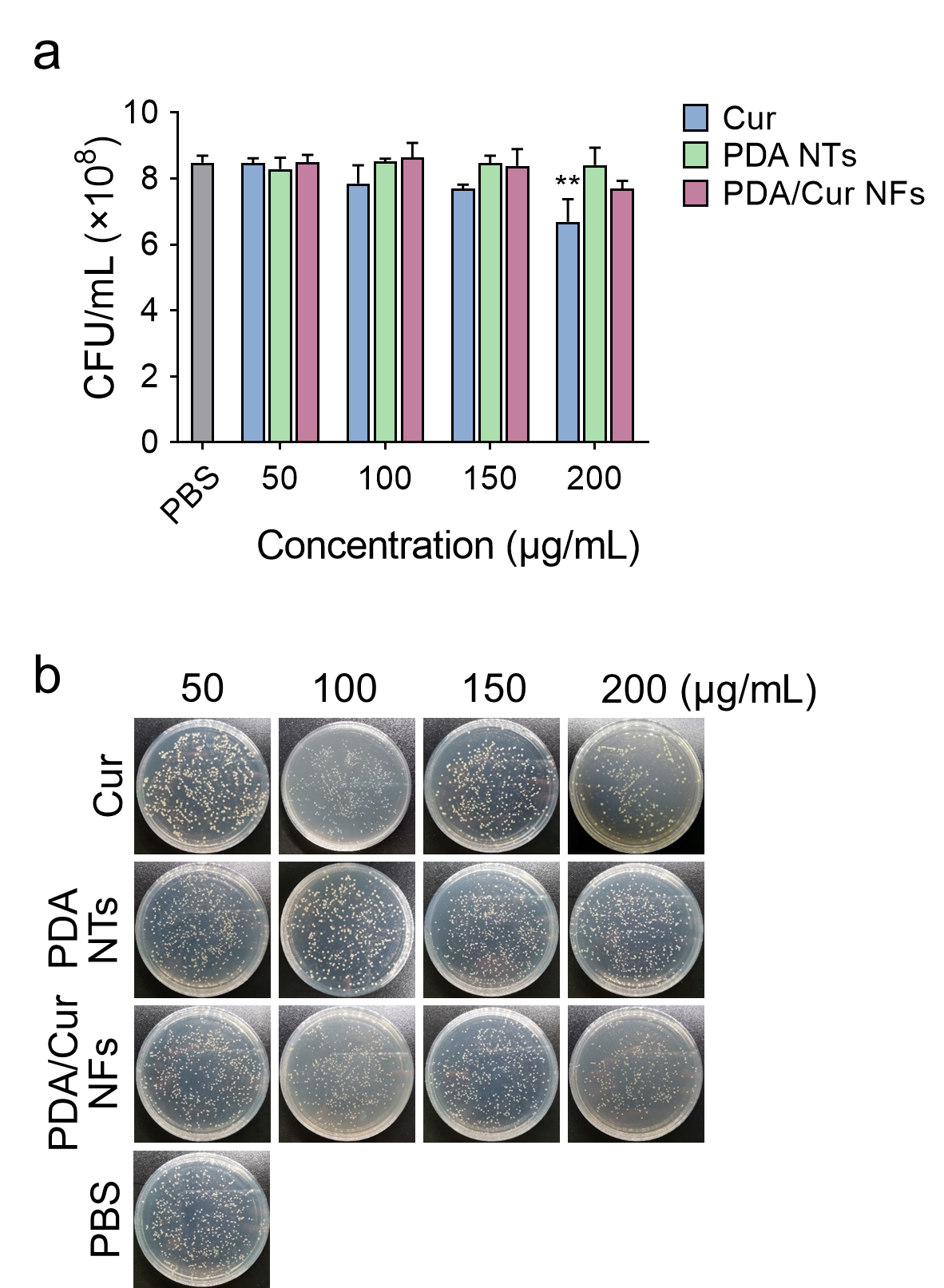


**Fig. S4** (a) Antibacterial effects of Cur nanocrystals, PDA NTs, and PDA/Cur NFs at different concentrations for 30 min incubation. (b) Photographs of bacterial colonies of MRSA after being treated with Cur nanocrystals, PDA NTs, and PDA/Cur NFs for 30 min at different concentrations.


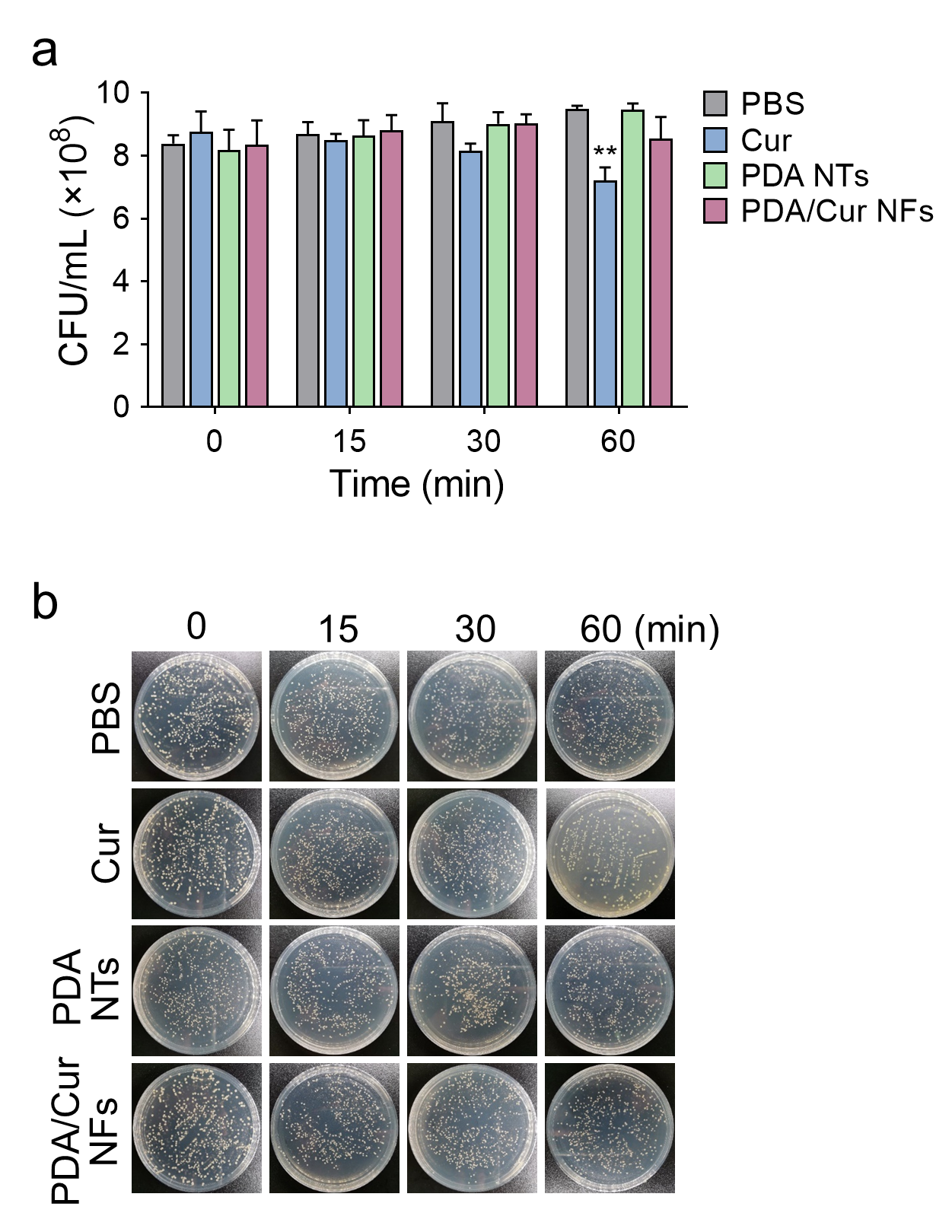


**Fig. S5** (a) Antibacterial effects of Cur nanocrystals (10 μg/mL), PDA NTs (90 μg/mL), and PDA/Cur NFs (100 μg/mL) for different incubation times. (b) Photographs of bacterial colonies of MRSA after being treated with Cur nanocrystals (10 μg/mL), PDA NTs (90 μg/mL), and PDA/Cur NFs (100 μg/mL) for different incubation times.


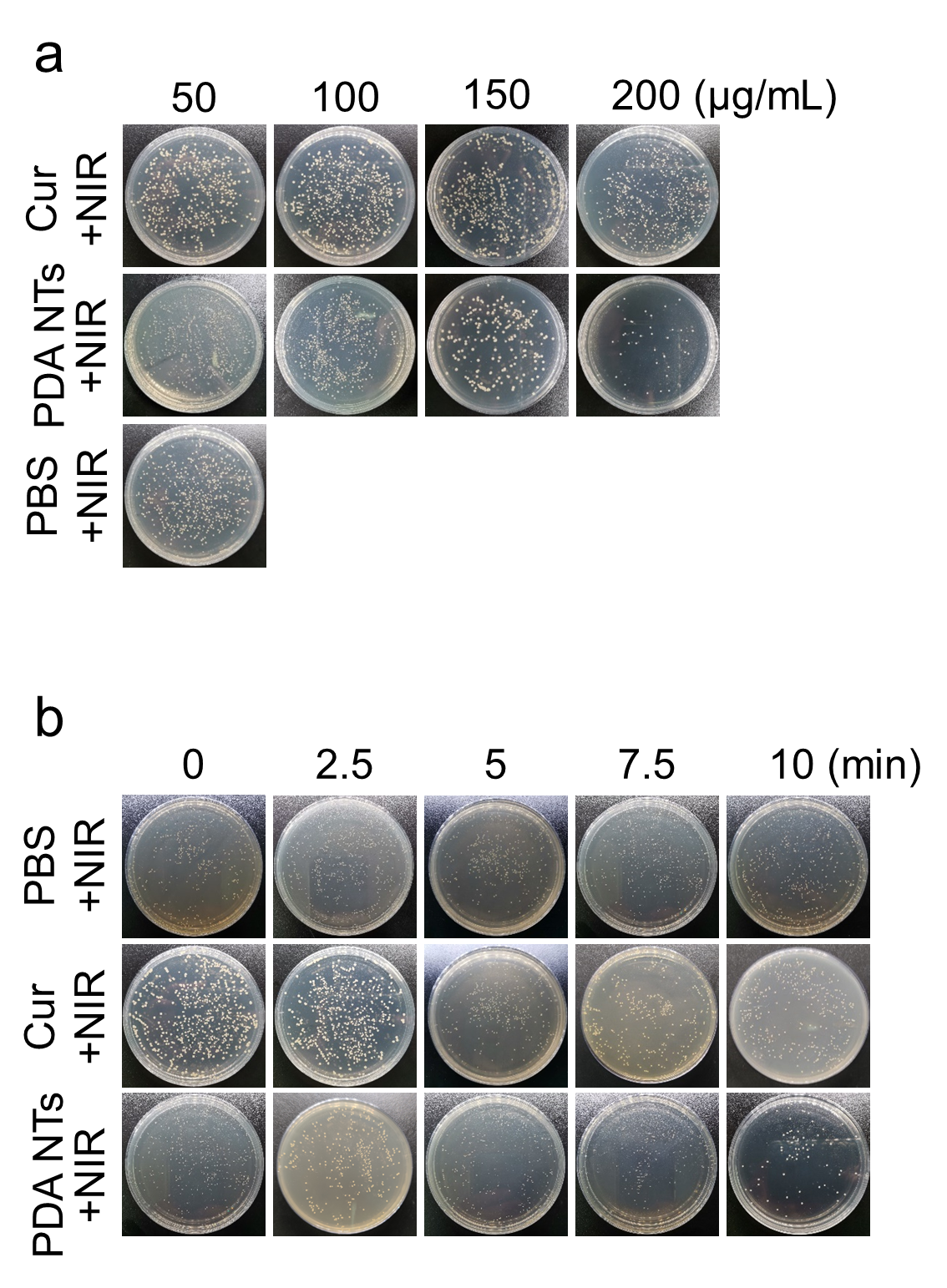


**Fig. S6** (a) Photographs of bacterial colonies of MRSA after being incubated with Cur nanocrystals and PDA NTs at different concentrations under NIR laser (808 nm, 0.8W/cm^2^, 300 s). (b) Photographs of bacterial colonies of MRSA after being treated with Cur nanocrystals (10 μg/mL) and PDA NTs (90 μg/mL) under NIR laser (808 nm, 0.8W/cm^2^) for different times.


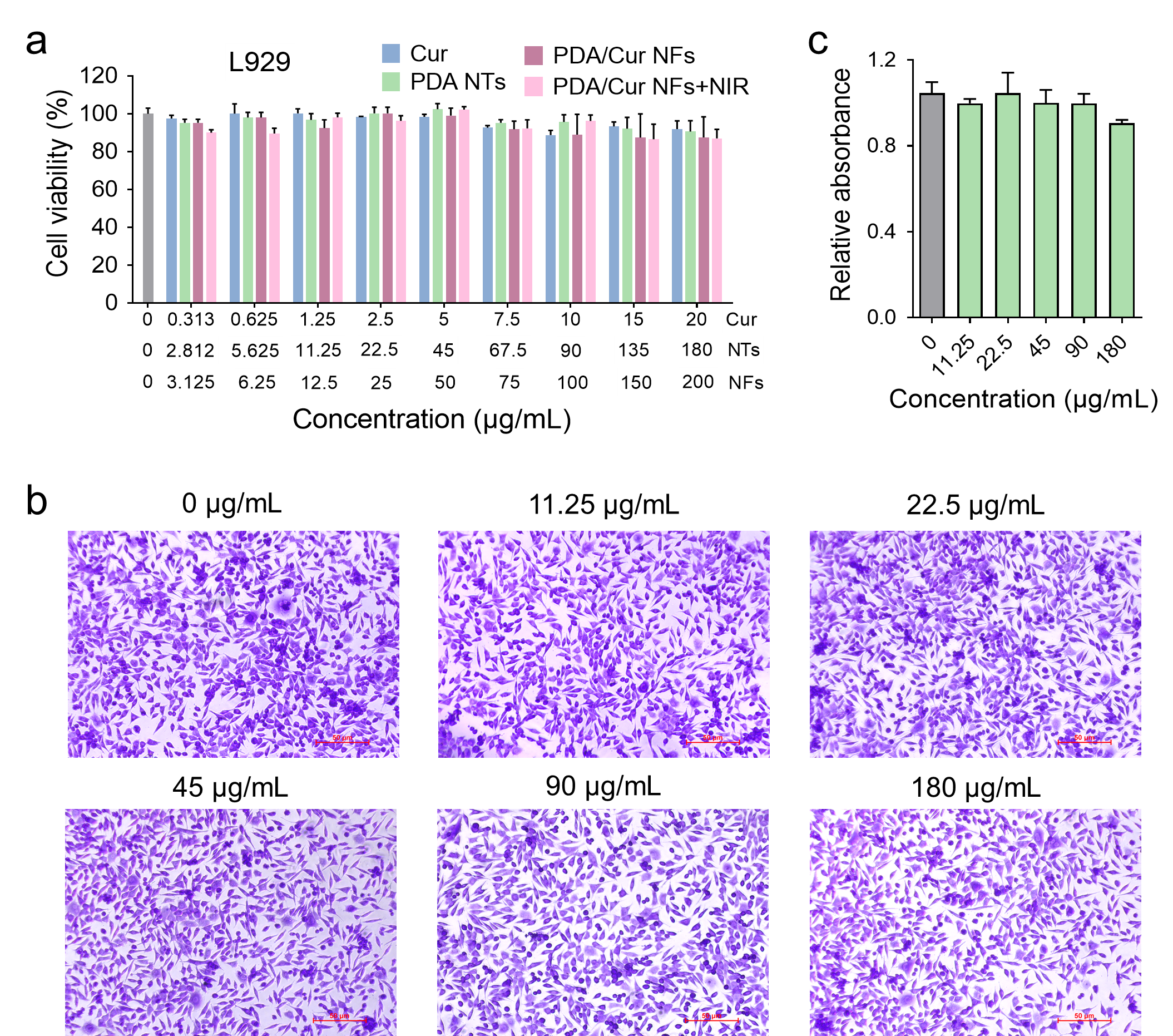


**Fig. S7** (a) The cell viability of L929 cell after being treated with Cur nanocrystals, PDA NTs, PDA/Cur NFs, and PDA/Cur NFs+NIR at 24 h post-incubation with different concentrations. (b)-(c) The crystal violet staining and relative absorbance of L929 cell after being treated with PDA NTs at 24 h post-incubation with different concentrations (scale bar = 50 μm).


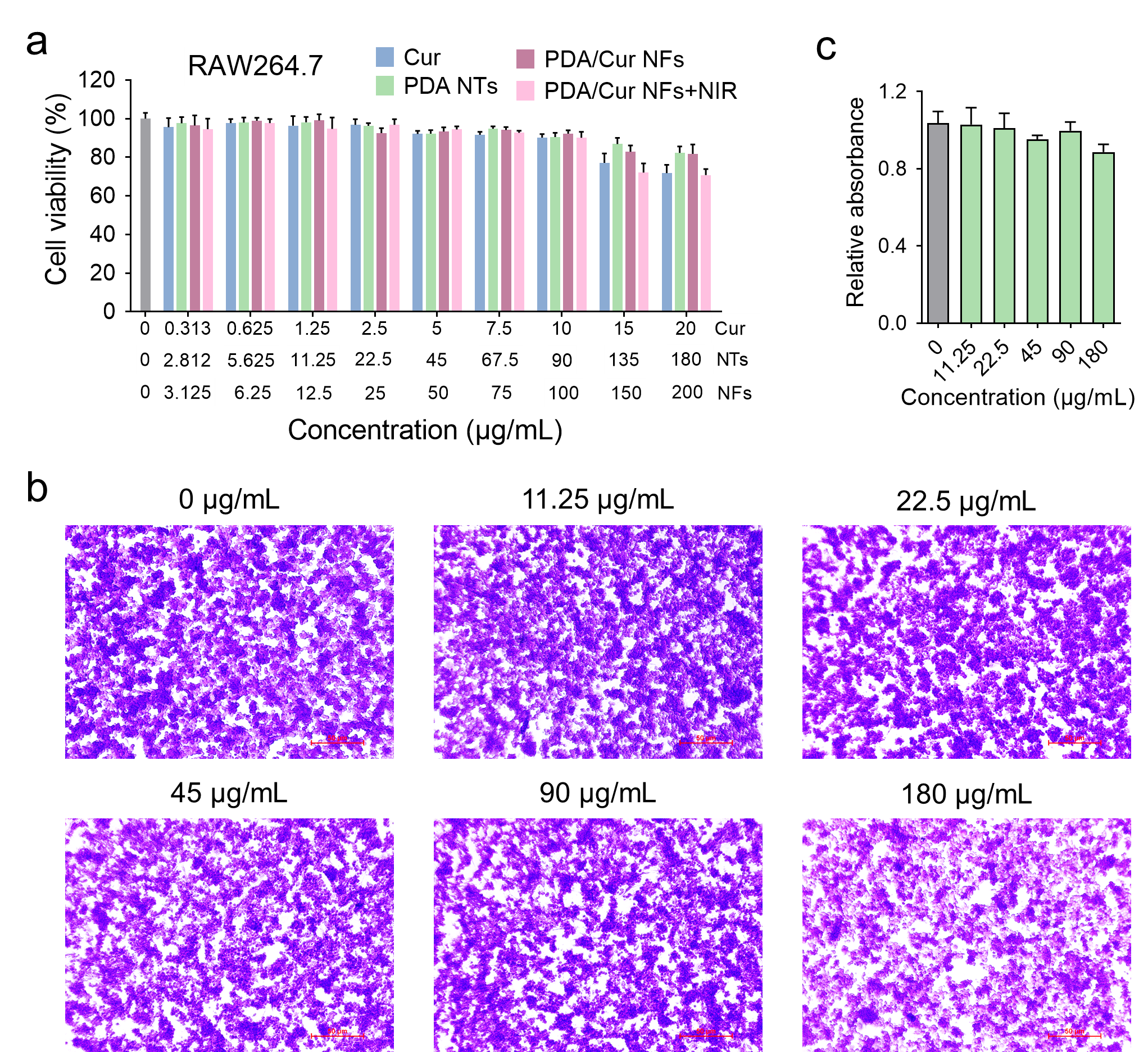


**Fig. S8** (a) The cell viability of RAW264.7 cell after being treated with Cur nanocrystals, PDA NTs, PDA/Cur NFs, and PDA/Cur NFs+NIR at 24 h post-incubation with different concentrations. (b)-(c) The crystal violet staining and relative absorbance of RAW264.7 cell after being treated with PDA NTs at 24 h post-incubation with different concentrations (scale bar = 50 μm).


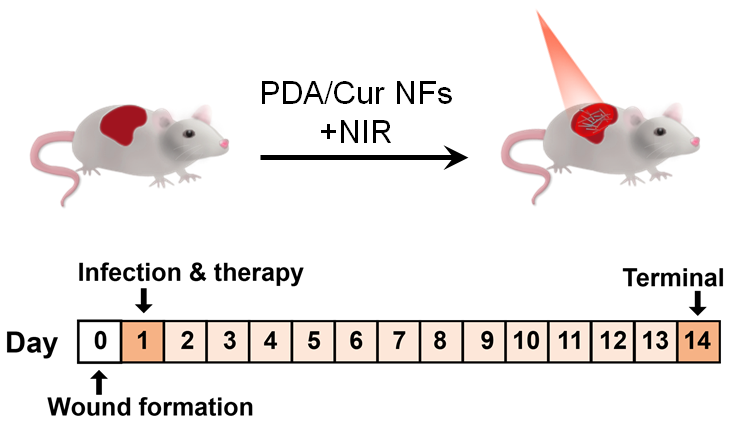


**Fig. S9** A schematic illustration showing the *in vivo* infected diabetic wounds at different time intervals under treatment.


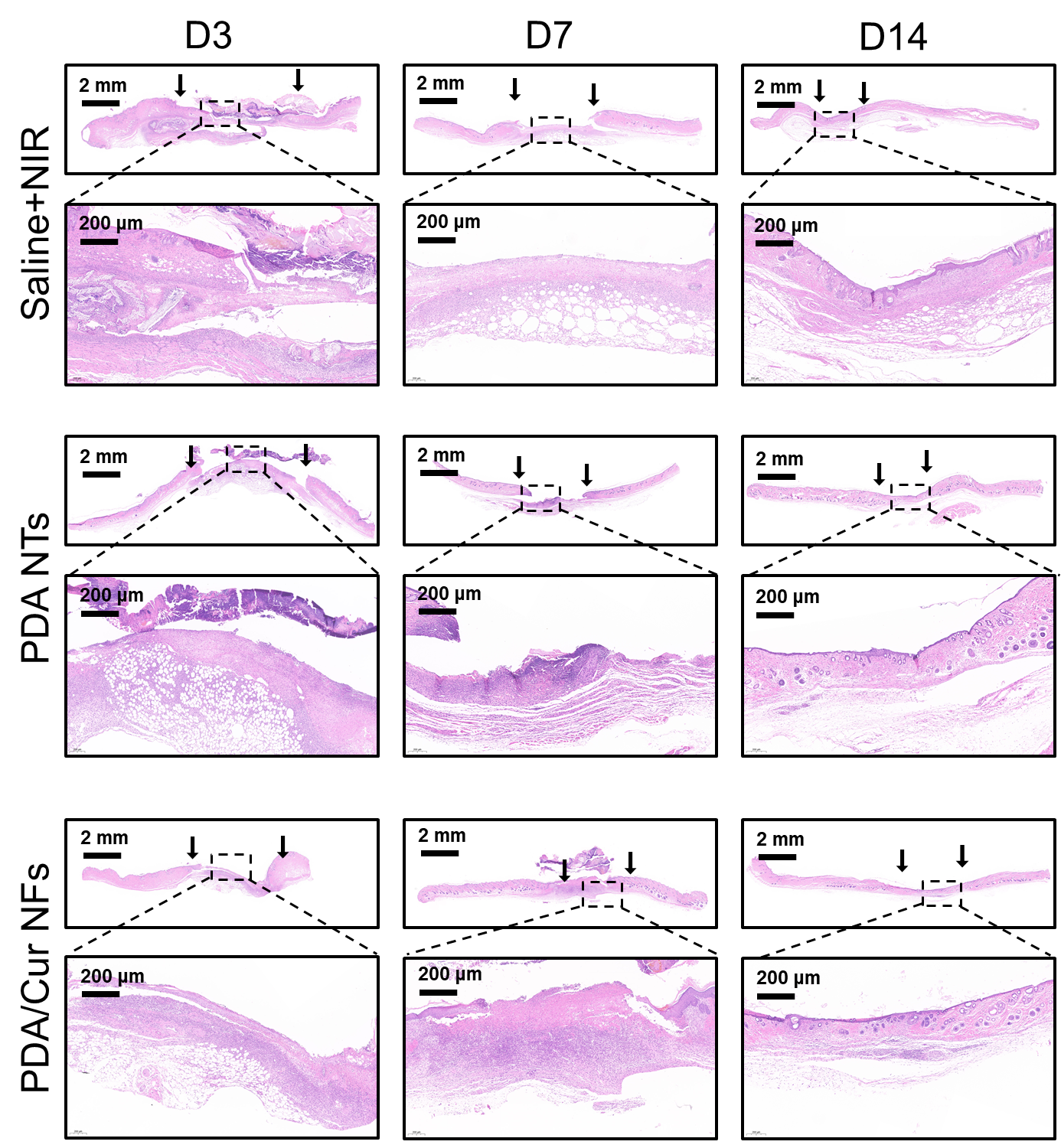


**Fig. S10** H&E staining of wounds treated with saline+NIR, PDA NTs, and PDA/Cur NFs on days 3, 7, and 14. Black arrows indicated the wound boundaries.


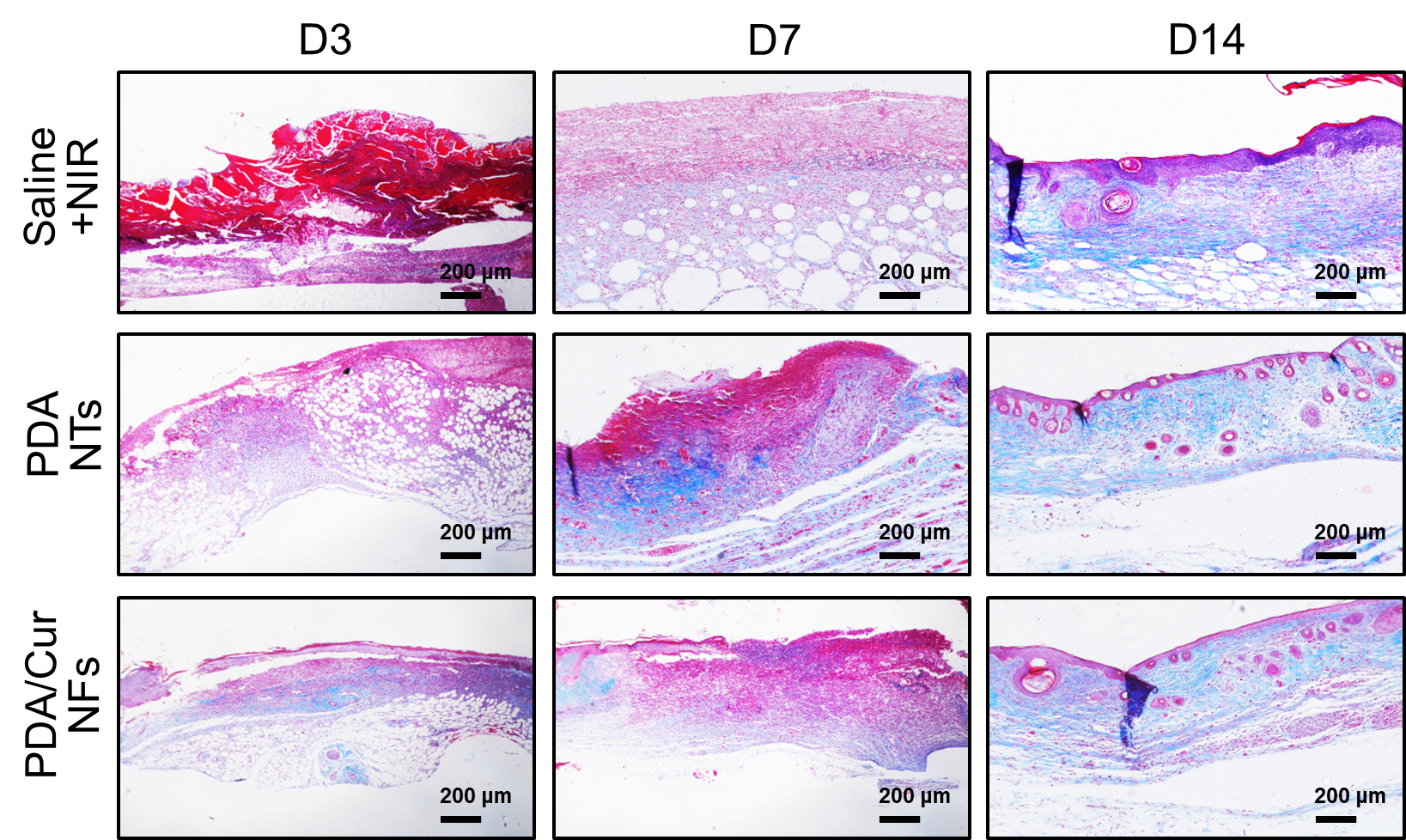


**Fig. S11** Masson staining of wounds treated with saline+NIR, PDA NTs, and PDA/Cur NFs on days 3, 7, and 14.


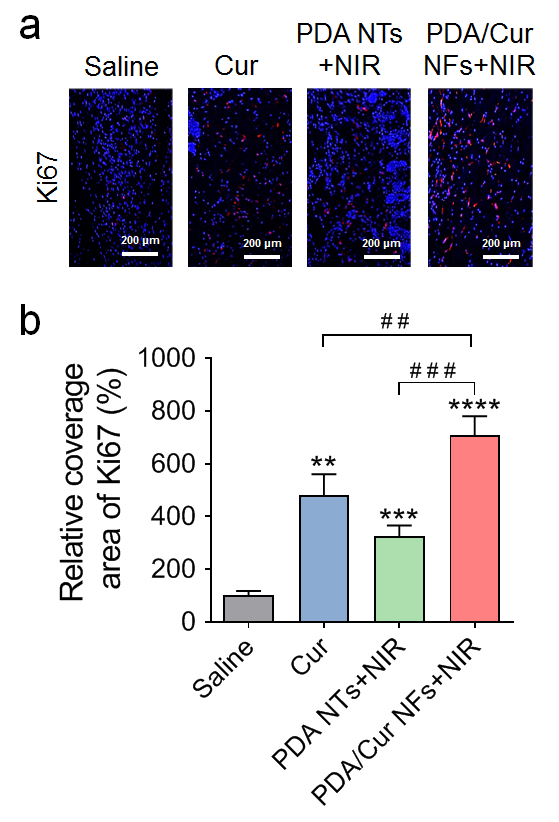


**Fig. S12** (a) Representative immunofluorescence images showing Ki67 for different groups. (b) The relative quantification of Ki67 after different treatments. Data are presented as mean ± SD (n = 3). ^**^*P* < 0.01, ^***^*P* < 0.001, ^****^*P* < 0.0001, vs saline group. ^##^*P* < 0.01, ^###^*P* < 0.001, vs the indicated groups.

a
